# Fourth-generation EGFR-TKI to overcome C797S mutation: past, present, and future

**DOI:** 10.1080/14756366.2025.2481392

**Published:** 2025-04-02

**Authors:** Die Zhang, Jumei Zhao, Yue Yang, Qiangfang Dai, Ning Zhang, Zhikuan Mi, Qianqian Hu, Xiaolong Liu

**Affiliations:** School of Medicine, Yan’an University, Yan’an City, China

**Keywords:** EGFRC797S mutation and triple mutation, the fourth generation EGFR-TKIs, antitumor activity

## Abstract

Overactivation of the epidermal growth factor receptor (EGFR) is prevalent in various tumours, rendering it a promising target for cancer therapy, particularly in the treatment of non-small cell lung cancer (NSCLC). Although the first through third generations of EGFR tyrosine kinase inhibitors (TKIs) have demonstrated significant efficacy, the emergence of drug resistance continues to pose a challenge. Current research is now focused on fourth-generation EGFR-TKIs, which specifically target the EGFR harbouring the C797S mutation. This review examines the design strategies, antitumor activity both *in vivo* and *in vitro*, binding modes, pharmacokinetics, as well as the advantages and disadvantages of each inhibitor, alongside the progress of clinical stage research related to fourth-generation inhibitors. Additionally, the review discusses future development directions for fourth-generation EGFR-TKIs, aiming to provide insights for successful research and development in this field.

## Introduction

Cancer is one of the leading causes of human death worldwide, and the global incidence and mortality of cancer are on the rise^1^^,^^2^. At present, chemotherapy is still the core method, supplemented by surgical resection and radiotherapy, and this method has considerable toxic and side effects^3^^,^^4^. Epidermal growth factor receptor (EGFR) plays a considerable role in the proliferation, survival, adhesion, migration, and differentiation of cancer cells, and is an effective target for cancer therapy^5^.

**Figure 1.**
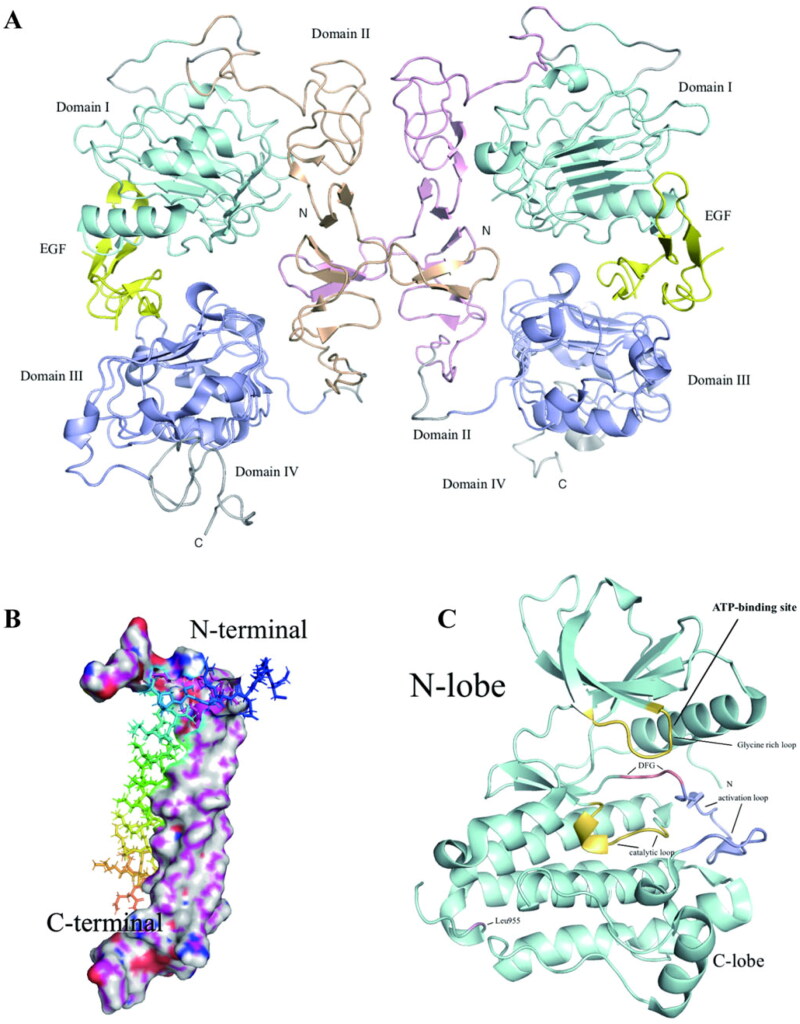
The extracellular domain of 2:2 EGF·EGFR complexes (PDB: 1IVO) (A). The structures of the EGFRTM dimer (PDB: 2JWA) (B). The EGFRK structures drawn from Protein Data Bank (PDB: 1M14) (C).

EGFR is a 170 kDa glycoprotein belonging to receptor tyrosine kinases. It has an extracellular ligand-binding domain, a hydrophobic transmembrane domain, and an intracellular domain with an ATP-binding active site on a tyrosine moiety (Figure 1)^6^. The extracellular domain contains 621 amino acids and is divided into four regions (exons 1–16). EGFR binds to epidermal growth factor (EGF) and undergoes homo- or heterodimerisation resulting in tyrosine phosphorylation, which is located in the intracellular tyrosine kinase domain^7^. The transmembrane domain contains 23 amino acids (Ile622 to Met644). The intracellular domain is divided into three regions: the flexible membrane-proximal region (exons 16 and 17), the tyrosine kinase domain (exons 18–24), and the C-terminal tail region (exons 25–28)^8^. The tyrosine kinase domain is divided into two parts: the N leaf and the C leaf. The ATP-binding active site is located between these two leaves. Kinase downstream signalling is activated by the trans-autophosphorylation interaction of the receptor N lobe with another receptor C lobe^9^. The kinase domain also contains lysine-rich residues, major sites for receptor ubiquitination^10^. In addition, the C-terminal tail contains several tyrosine residues that undergo autophosphorylation upon receptor activation. This triggers several downstream signalling cascades, including RAS/RAF/ERK, STAT, and PI3K/AKT/mTOR pathways, which subsequently produce numerous effects in the cytoplasm, such as cell proliferation, differentiation, migration, growth, and apoptosis inhibition^11^. Adenosine diphosphate (AMPPNP) is a hydrolysable form of ATP whose crystal structure (PDB: 3VJO) is linked to wild-type EGFR (EGFRWT). AMPPNP thus has typical hydrogen bonds with Met793 and Gln791, which activate downstream signalling and cascade processes in the cytoplasm, including cell growth and apoptosis inhibition. The small molecule EGFR-tyrosine kinase inhibitor (TKI) functions by reversibly competing with ATP for binding to the intracellular tyrosine kinase domain of EGFR, reducing EGFR autophosphorylation and downstream signalling.

Drug development focused on EGFR is a key area in anti-tumour research^12^. EGFR-TKIs from different generations, including gefitinib, afatinib, and osimertinib, have been approved for cancer treatment^13^^,^^14^, leading to improved objective response rates and extended progression-free survival in patients, demonstrating positive therapeutic outcomes^15^.

**Figure 2.**
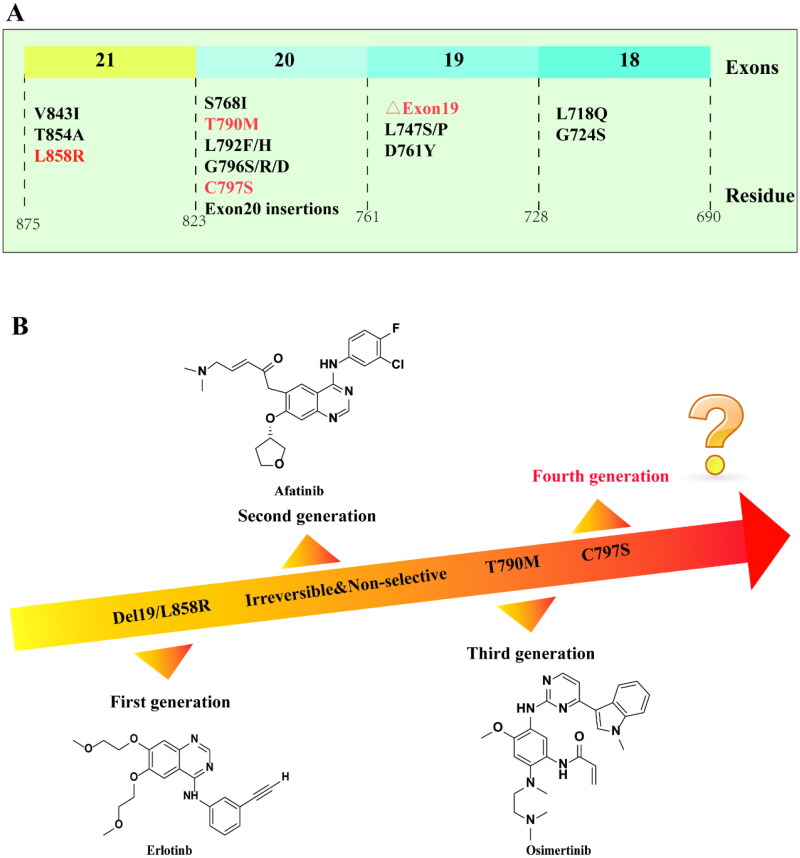
EGFR common mutation (A) and representative EGFR-TKIs (B).

However, during use, the researchers found a drug-resistance mutation at the target, which could divide into primary and acquired types^16^^,^^17^. Primary drug resistance results from gene mutation^18^, EGFR gene located on the short arm of chromosome 7, which is a common mutation in exon 18–21 during treatment^19^. The most common EGFR mutations in non-small cell lung cancer (NSCLC) patients include L858R point mutations in exon 21 and deletion of exon 19^20^ (Figure 2). These mutations lead to enhanced EGFR signal transduction and promote cancer development, but the first generation of EGFR-TKIs has a significant therapeutic effect^21^. On the basis of primary drug resistance, the second and third gene mutations are called acquired drug resistance^22^. Among them, the second mutation mainly appeared at the T790M site in exon 20, and threonine replaced methionine^23^. The second-generation EGFR-TKIs have been developed to target this mutation. Due to the evident off-target effect and extreme side effects of the second-generation EGFR-TKIs, the researchers developed the third-generation EGFR-TKIs^24^ (Figure 2(B)). EGFR C797S mutation arises during the treatment process, with serine SER797 replacing cysteine CYS797^25^. To overcome the triple mutation including C797S, the fourth generation EGFR-TKIs are being developed.

## Research progress of C797S drug resistance mutant EGFR-TKIs

This paper reviews the preclinical and clinical research progress of different structural types of EGFR-TKIs targeting C797S mutation.

### Progress in preclinical study of C797S resistance mutant EGFR-TKIs

The first and second generation EGFR-TKIs with quinazoline scaffolds, such as gefitinib and erlotinib^26^, the third generation EGFR-TKIs based on pyrimidine skeletons, such as osimertinib^27^, and ALK inhibitor such as brigatinib, etc. The structural optimisation of these excellent compounds is an effective way to develop fourth generation EGFR-TKIs against C797S mutation^28^^,^^29^.

#### Hybrid derivatives of gefitinib and erlotinib

Wakte and coworkers^30^ synthesised a series of efficient molecules using similar scaffolds with different atoms in gefitinib and erlotinib. The optimal compound **1** (Figure 2) exhibited strong inhibitory against EGFR^L858R/T790M/C797S^ and showed significant inhibition against HCC827, A549, and HT29. Compared with the control group, it significantly induced early and late apoptosis.

**Figure 3.**
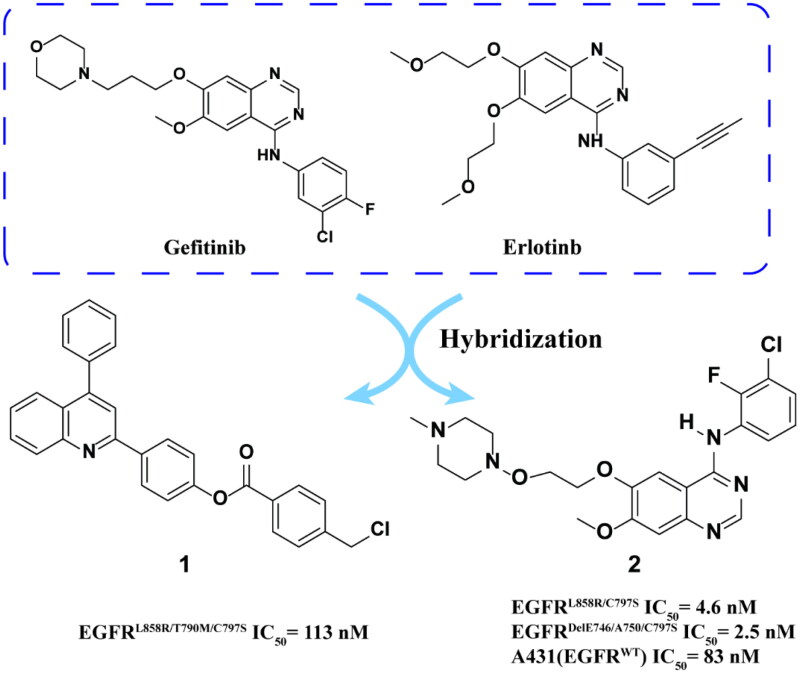
Chemical structures of gefitinib and erlotinib hybrid derivatives **1–2** and antitumor activity.

Crich and coworkers^31^ reported a highly selective, orally bioavailable, brain penetrating EGFR-TKI **2** containing trisubstituted hydroxylamine groups (Figure 3), used in the treatment of central nervous system metastasis of NSCLC. This compound demonstrated remarkable efficacy against drug-resistant cell lines, specifically EGFR^L858R/C797S^ and EGFR^DelE746/A750/C797S^. In A431 cells, which exhibit high levels of EGFRWT expression, the selectivity of compound **2** was found to be nearly 12–33 times greater than that of wild-type EGFR. Furthermore, compound **2** exhibited favourable pharmacokinetic properties, including good exposure and an acceptable half-life in Sprague-Dawley (SD) rats. Notably, in an intracranial patient-derived xenograft (PDX) mouse model, significant tumour shrinkage was observed following oral administration. These findings suggest that the introduction of trisubstituted hydroxylamine moieties may enhance drug performance while preserving exceptional biological activity.

**Figure 4.**
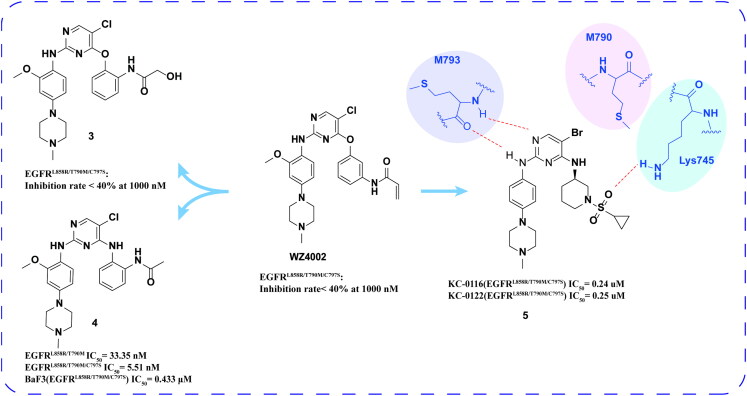
Chemical structures of WZ4002 derivatives **3–5** and antitumor activity.

Romu et al.^32^ connected various electrophilic and non-electrophilic groups to phenoxy groups to synthesise a series of WZ4002 derivatives. Among them, the optimal compound **3** (Figure 4) effectively inhibited EGFR^L858R/T790M/C797S^, and the inhibition rate was 90% at the concentration of 1000 nM.

Xu and coworkers^33^ developed a series of ortho-amidophenylaminopyrimidines compounds according to WZ4002. The optimal compound **4** (Figure 3) showed intense inhibitory activity on EGFR^L858R/T790M^, EGFR^L858R/T790M/C797S^, and BaF3 (EGFR^L858R/T790M/C797S^) and low toxicity in human normal liver cell L02. Compound **4** has stronger activity and better selectivity to inhibit the three mutants, obviously induces late apoptosis in a dose-dependent manner and has higher safety *in vivo*. Furthermore, compound **4** functions not only as a competitive ATP inhibitor but also as an allosteric inhibitor of EGFR^L858R/T790M/C797S^.

Zhang and coworkers^34^ designed and synthesised a series of 2-amino pyrimidines based on the lead compounds. Compound **5** (Figure 4) showed micromolar inhibitory activity against triplet mutant KC-0116 and KC-0122 cells. Compared with brigatinib, these compounds show more prominent and selective inhibitory effects. Docking studies show that **5** forms hydrogen bonds with MET793 residues and LYS745 residues in addition to two important hydrogen bonds. This is an important reason why compound **5** has strong inhibitory activity against EGFR^Del19/T790M/C797S^. In addition, its bromine atoms interact with van der Waals force towards MET790, which may be the key factor for its mutation selectivity to EGFR^WT^.

#### Afatinib, vandetanib, and avitinib derivatives

**Figure 5.**
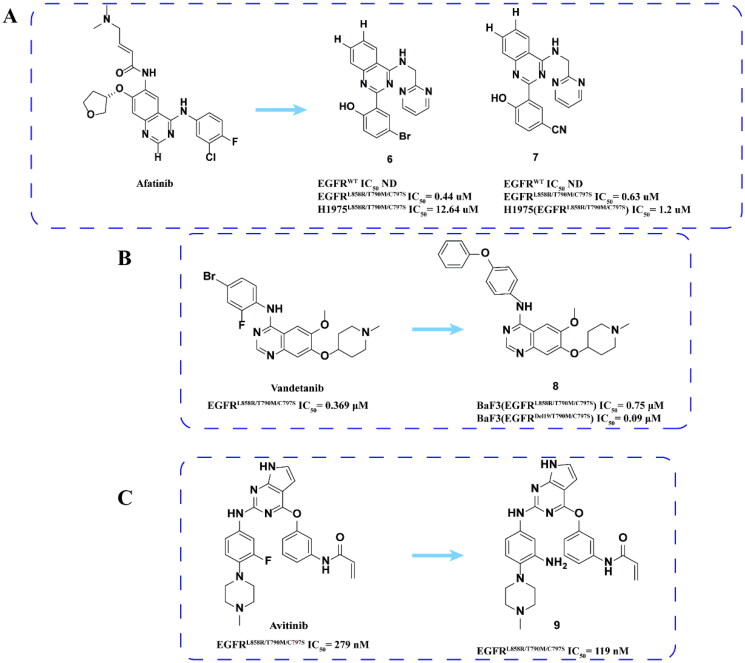
Chemical structures of alphatinib derivatives **6–7** and antitumor activity (A). Chemical structure of vandetanib derivatives **8** and antitumor activity (B). Chemical structure of avetinib derivatives **9** and antitumor activity (C).

Wang and coworkers^35^ synthesised a series of 2-aryl-4-aminoquinazoline compounds to resist EGFR^L858R/T790M/C797S^ mutation based on afatinib. The activity assay showed that compounds **6** and **7** (Figure 5) were effective inhibitors of EGFR^L858R/T790M/C797S^. However, its activity against H1975 (EGFR^L858R/T790M/C797S^) is poor. In addition, the drug showed good stability in liver microsomes, but low oral bioavailability.

The IC_50_ value of the second-generation EGFR inhibitor vandetanib to EGFR^L858R/T790M/C797S^ is 0.369 μM^36^^,^^37^. On this basis, Li and coworkers^38^ designed a series of 4-aniline quinoline compounds that occupy both ATP binding bag and allosteric site against three mutations. Compound **8** (Figure 5(B)) has a good anti-proliferation effect and has an obvious inhibitory effect in BaF3 (EGFR^Del19/T790M/C797S^) cells in a dose-dependent manner. **8** can also significantly inhibit the tumour growth of the BaF3 (EGFR^Del19/T790M/C797S^) xenotransplantation model. The pharmacokinetic results showed that the half-life and bioavailability of **8** were general. In summary, **8** is an effective new EGFR^C797S^ inhibitor with both ATP binding pocket and allosteric site. However, its pharmacokinetic effect deserves further improvement.

He and coworkers^39^ designed a series of pyrrolo[2,3-d]pyrimidine derivatives by modifying the amino groups of a 2-phenylamino benzene ring and pyrrole ring based on avitinib. The inhibitory activity indicated that compound **9** (Figure 5(C)) had an effective inhibitory effect on EGFR^L858R/T790M/C797S^, which was slightly higher than that of avetinib.

#### P38 kinase inhibitor derivatives

**Figure 6.**
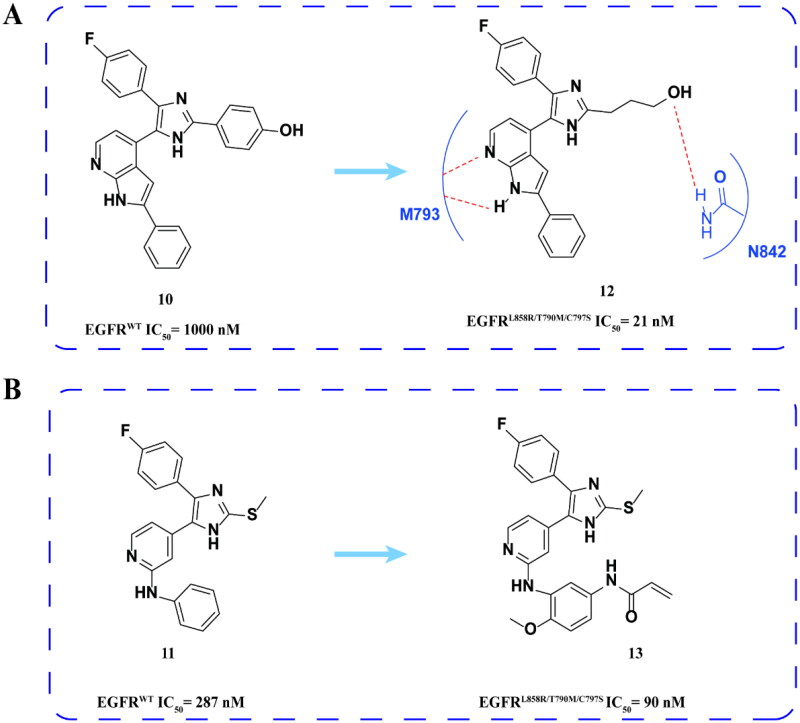
Chemical structures of P38 inhibitor **10** and its derivatives **11–13** and antitumor activity.

Laufer and coworkers^40^ confirmed the non-target effect of EGFR inhibition at compound **10** (Figure 6) in the study of p38 inhibitors containing imidazole skeleton. Later, the group screened about 2000 compounds with structures like **10**. Among these compounds, **11** (Figure 6) was confirmed by activity verification that it can be used as a lead compound for further optimisation. Then, the group optimised the two substituents of **10** imidazole rings and the substituents of **11** phenylaminobenzene rings. The results showed that compounds **12** and **13** (Figure 6) had formidable inhibitory activity on EGFR^L858R/T790M/C797S^. The results of docking showed that the pyrrolo[2,3-b] pyridine part of **12** formed a bidentate hydrogen bond with M793 in the hinge region of EGFR^T790M^. Hydroxyl groups form additional hydrogen bonds with N842, which may be the reason for the increase in inhibitory activity.

**Figure 7.**
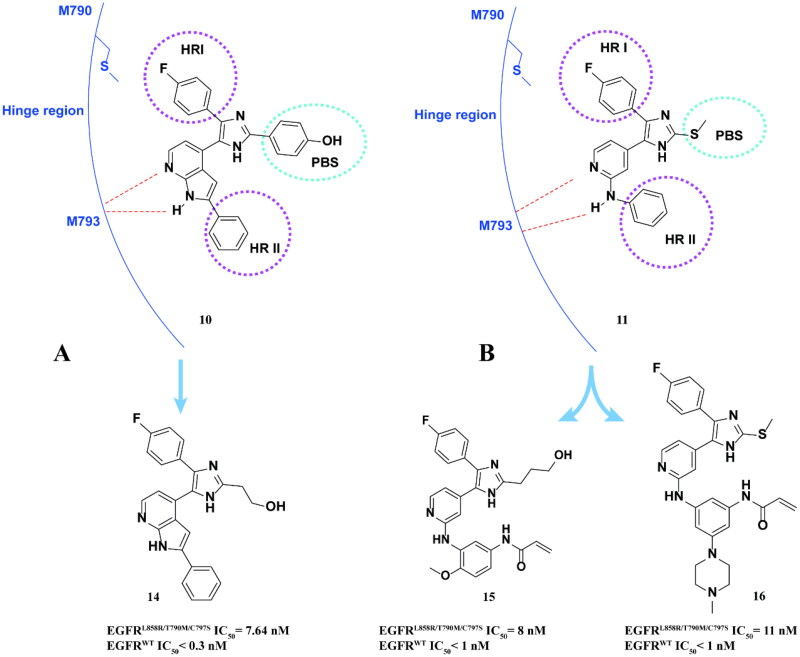
Chemical structures of **10** and its derivatives **14** and antitumor activity (A). Chemical structures of **11** and its derivatives **15–16** and antitumor activity (B).

Subsequently, the team continued to study the structure–activity relationship of lead compounds **10** and **11** (Figure 7) to EGFR mutants, changing the hydrophobic group I (HRI), hydrophobic group II (HRII), and phosphate binding site (PBS) of EGFR kinase domain^41^^,^^42^. Finally, compounds **14**, **15**, and **16** (Figure 7) against EGFR^L858R/T790M/C797S^ were screened by activity. However, these compounds also showed high inhibitory activity against EGFR^WT^.

In summary, trisubstituted imidazole is an effective framework for the development of the fourth generation EGFR-TKIs, but its selectivity also needs to be further improved.

#### Osimertinib derivatives

**Figure 8.**
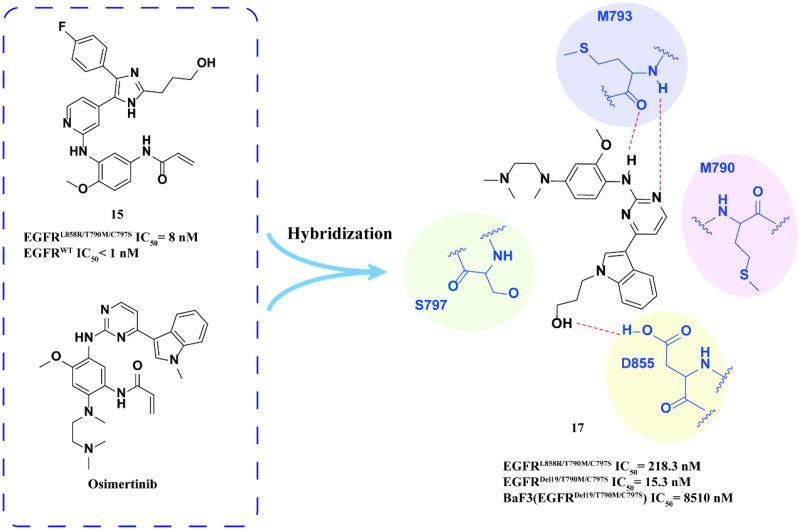
The binding mode of derivative **17**.

Zhao and coworkers^43^ reported that compound **15** (Figure 8) formed a hydrogen bond between its hydroxyl group and the side chain of D855 of the DFG motif. The optimal compound **17** (Figure 8), was obtained by attaching the indole moiety of osimertinib with a hydroxyalkyl chain. Compound **17** exhibited strong inhibition against EGFR^Del19/T790M/C797S^ and moderate inhibition activity against EGFR^L858R/T790M/C797S^. It also displayed weak anti-proliferation activity against BaF3 (EGFR^Del19/T790M/C797S^), induced apoptosis in BaF3 (EGFR^Del19/T790M/C797S^) and arrested the cell cycle in the G1 phase. Docking studies revealed that the addition of an alkyl chain to the hydroxyl-terminated structural unit in osimertinib scaffold allowed compound **17** to form a hydrogen bond with Asp855 at the conservative DFG site, thereby enhancing its inhibitory activity against EGFR^L858R/T790M/C797S^.

**Figure 9.**
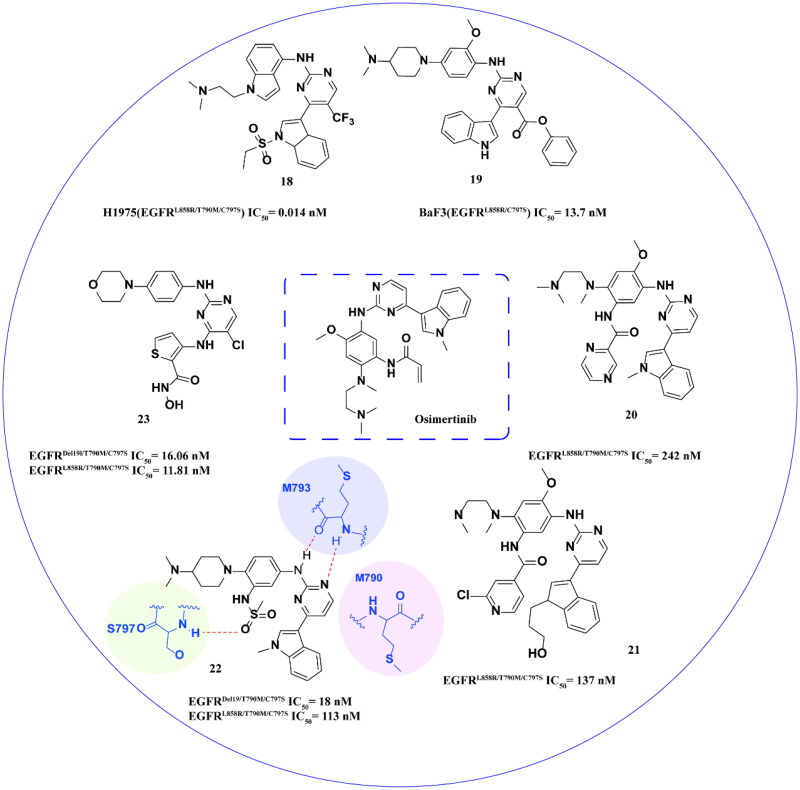
Chemical structures of osimertinib derivatives **18–23** and antitumor activity.

Yang and coworkers^44^ utilised a cyclisation strategy to enhance the interaction between compounds and mutants based on osimertinib, resulting in the synthesis of the optimal compound **18** (Figure 9). Compound **18** exhibited positive inhibitory activity and high selectivity towards NSCLC H1975 (EGFR^L858R/T790M/C797S^). *In vivo* experiments demonstrated that compound **18** had superior antitumor efficacy compared to the positive controls TQB3804 and brigatinib, leading to a significant inhibition of tumour progression (TGI = 70.75%) at higher doses. Pharmacokinetic analysis revealed a half-life of 3.67 h and a *C*_max_ value of 130 ng/mL for compound **18**. The oral half-life was determined to be 5.37 h, with a moderate bioavailability (*F* = 28.07%). Overall, compound **18** exhibited favourable pharmacodynamic properties, safety profile, and desirable pharmacokinetic characteristics.

Rauh and coworkers^45^ developed a series of highly efficient fourth-generation reversible EGFR inhibitors with osimertinib as the core structure. The optimal compound **19** demonstrated an IC_50_ of 13.7 nM towards BaF3 (EGFR^L858R/C797S^) and 1.8 μM towards A431 (EGFR^WT^), as illustrated in Figure 8. This compound exhibited good selectivity.

Chen and coworkers^46^ designed a series of osimertinib derivatives that do not contain acrylamide groups. Among these, compounds **20** and **21** (Figure 9) exhibited promising inhibitory activity and low cytotoxicity. Compound **21**, in particular, showed significant inhibitory activity and selectivity towards EGFR^L858R/T790M/C797S^, with potential kinase selectivity that could mitigate side effects associated with excessive inhibition of EGFR^WT^. However, the lack of pharmacokinetic data for both compounds hinders further experiments.

Zhang and coworkers^47^ synthesised phenylamino-pyrimidine compounds using osimertinib as a scaffold. Following optimisation, compound **22** (Figure 9) exhibited potent inhibitory activity against EGFR^Del19/T790M/C797S^ and EGFR^L858R/T790M/C797S^, demonstrating significant inhibitory effects in KC-0116 (EGFR^Del19/T790M/C797S^) and KC-0122 (EGFR^L858R/T790M/C797S^). Furthermore, compound **22** dose-dependently inhibited EGFR phosphorylation in KC-0116 cells, leading to cell cycle arrest in the G1 phase and induction of apoptosis. **22** showed a strong antitumor effect in the KC-0116 cell transplantation tumour model in nude mice. Molecular docking analysis revealed that the oxygen atom of the methanesulfonamide group in compound **22** formed an additional hydrogen bond with SER797, distinguishing it from the interaction of osimertinib with the triple mutant EGFR. This differential binding mechanism likely contributes to the potent effects of compound **22** on both enzyme and cellular activities.

Yan and coworkers^48^ developed a series of pyrimidine compounds with high efficiency containing hydroxamic acid fragments based on the infrastructure of osimertinib. The optimal compound **23** (Figure 9) had a better inhibitory effect on EGFR^Del19/T790M/C797S^ and EGFR^L858R/T790M/C797S^, which was significantly higher than that of osimertinib. In addition, **23** can inhibit colony formation, block cells in the G2 phase, and inhibit cell migration. The *in vivo* transplantation model in mice showed that **23** had an apparent inhibitory effect on tumour regression and had no obvious toxicity.

#### Brigatinib derivatives

**Figure 10.**
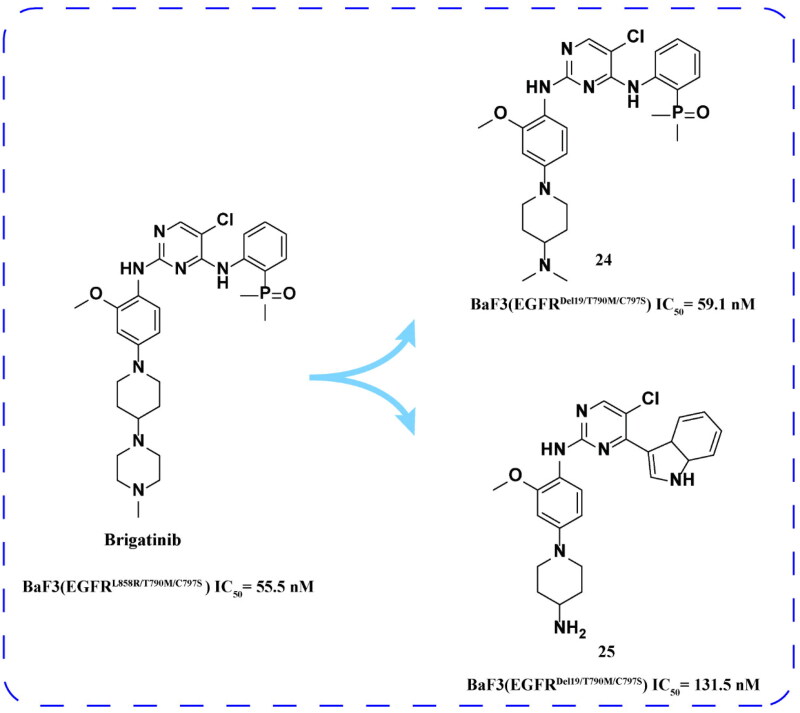
Chemical structures of brigatinib and derivatives **24–25** and antitumor activity.

Brigatinib (Figure 10) is an oral, effective, and selective double-target TKI of anaplastic lymphoma kinase (ALK) and c-ros oncogene 1 (ROS1). It is approved for the treatment of advanced NSCLC with positive ALK^49^. The IC_50_ of the drug against ALK and FLT3 mutants was 6.6 nM, and the IC_50_ for EGFR^L858R^ was 1.5 nM^50^.

Brigatinib binds to the ATP attachment site of ALK by adopting a U-shaped conformation. Its potency against ALK is attributed to chemical substituents introduced on the bis aniline pyrimidine backbone^51^^,^^52^. Another unique structural feature is the phosphine oxide group (DMPO) moiety, which is attached to the NH_2_ group of the benzene ring at carbon 4. Brigatinib’s selective profile relative to ALK is attributable only to the DMPO moiety, whose chemical profile determines extended fluid solubility, reduced lipophilicity, and reduced protein binding^53^. In addition to favourable ADME properties, it can also improve cell potency and selectivity. In the docked EGFR-brigatinib model, the phosphine oxide group DMPO completely occupies the triphosphate binding space of the ATP binding site, and the electrostatic or van der Waals interaction energy of all atoms in this group is significantly increased, indicating that brigatinib has a specific interaction with EGFR and helps to improve its efficacy against EGFR triple mutations. The presence of DMPO moiety improves its potency by about 70-fold compared with other unsubstituted analogs^54^^,^^55^. Brigatinib binding poses also indicate that there appears to be sufficient room for substitution on the piperidine ring and benzene ring attached to the phosphine oxide group. These two functional groups may be suitable for partial modification to enhance EGFRC797S/Del19 and EGFRC797S/T790M/Del19 binding affinity^56^.

Katayama and coworkers^57^ have shown that brigatinib can effectively inhibit the triple mutation of EGFR^L858R/T790M/C797S^ *in vitro* and *in vivo*. Its IC_50_ for BaF3 (EGFR^L858R/T790M/C797S^) is 55.5 nM (Figure 10). The affinity of brigatinib to EGFR^L858R/T790M/C797S^ was 10 times higher than that to EGFR^WT^, which decreased the phosphorylation of EGFR and its downstream signal pathway in a dose-dependent manner and inhibited the growth of PC9 (EGFR^Del ^^19^) and PC9 (EGFR^Del19/T790M^) or PC9^(EGFRDel19/T790M/C797S)^. In addition, combining with cetuximab can reduce the EGFR. After that, the team tested several other ALK-TKIs on the BaF3 (EGFR^Del19/T790M/C797S^) based on the structure of brigatinib. Among them, compounds **24** and **25** (Figure 9) have highly inhibitory effects on BaF3 (EGFR^Del19/T790M/C797S^).

Gou and coworkers^58^ designed and synthesised a range of novel aniline derivatives based on this lead compound. The optimal compound **26** (Figure 10) showed a strong antiproliferative effect on BaF3 (EGFR^Del19/T790M/C797S^), EGFR^Del19/T790M/C797S^, and EGFR^L858R/T790M/C797S^. Compared with EGFR^WT^, the selectivity of aniline derivatives to EGFR^Del19/T790M/C797S^ is more than 50 times. *In vivo*, **26** could inhibit the growth of PC9, BaF3, and xenograft tumour models with high EGFR^Del18/T790M/C797S^ expression in a dose-dependent manner. In addition, the pharmacokinetic level showed that **26** had higher exposure concentration and plasma concentration and longer half-life. Compound **26** showed positive inhibitory activity and high selectivity *in vivo* and *in vitro*. Pharmacokinetic results and toxicity tests showed that the compound might be used for further clinical trials.

**Figure 11.**
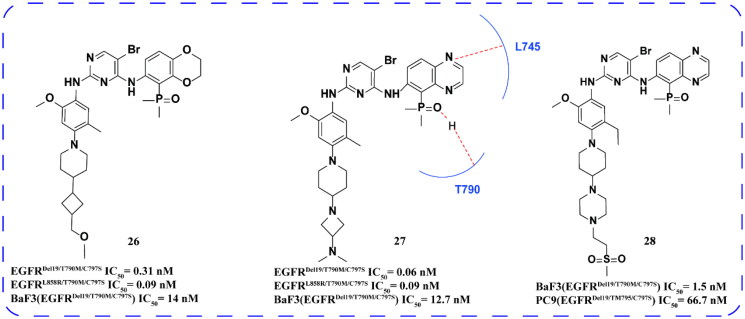
Chemical structures of brigatinib derivatives **26–28** and antitumor activity.

The team^59^ synthesised a series of hydroxyquinazoline phosphate compounds that displayed potent inhibitory activity against EGFR^C797S^ and high selectivity for EGFR^WT^ on the brigatinib skeleton. The optimal compound **27** (Figure 11) exhibited strong anti-proliferative activity against BaF3 cells with high expression of EGFR^Del19/T790M/C797S^, EGFR^Del19/T790M/C797S^, and EGFR^L858R/T790M/C797S^. Additionally, these compounds showed low inhibitory activities against EGFR^WT^ and A431 cells. Compound **27** demonstrated favourable pharmacokinetics and antitumor activity in mice with PC9 (EGFR^Del19/T790M/C797S^). Docking studies revealed that the oxygen atom on the phosphoryl group in these compounds formed a robust hydrogen bond with THR790, while the quinoxaline ring N bound to the hydrogen bond of LYS745, thereby enhancing the inhibitory effect on EGFR^C797S^. Consequently, compound **27** exhibited superior inhibitory activity compared to brigatinib, displaying high selectivity and efficacy *in vivo*, warranting further investigation.

Xie and coworkers^60^ utilised brigatinib as the foundation to synthesise the optimal compound **28**. Compound **28** exhibited significantly higher inhibitory activity against EGFR^L858R/T790M/C797S^ and EGFR^Del19/T790M/C797S^ compared to brigatinib. Moreover, it displayed a notable selectivity for EGFR^WT^. Pharmacokinetic analysis revealed that the *in vivo* clearance of compound **28** was approximately 2.4 L/h/kg, indicating moderate stability, favourable oral bioavailability, and high plasma exposure levels. These findings suggest that the compound possesses promising tumour inhibitory properties and is well-suited for future *in vivo* research via oral administration.

**Figure 12.**
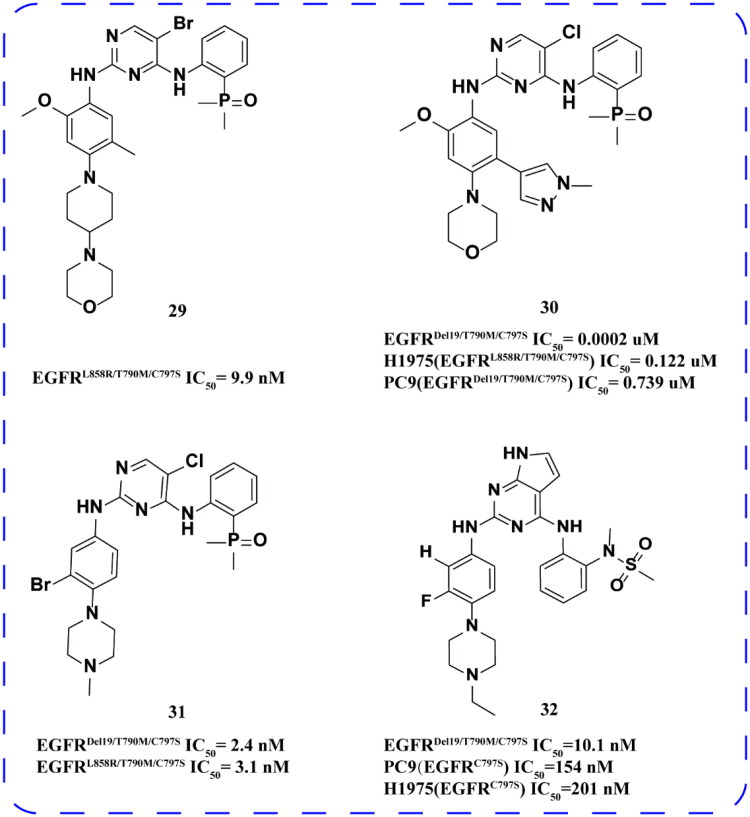
Chemical structures of brigatinib derivatives **29–32**.

Zhu and coworkers^61^ also designed and synthesised sequence 2-amine-4-oxophosphamide compounds using this lead compound. The optimal compound **29** (Figure 12) had outstanding inhibitory activity on H1975 (TL) and H1975 (CTL) cancer cells and the EGFR^L858R/T790M/C797S^. The half-life (*t*_1/2_) and intrinsic clearance (CLint) parameters of compound **29** indicated good metabolic stability, suggesting its potential as an oral drug candidate for further investigation.

Zhao and coworkers^62^ through the modification of Brigatinib, the optimal compound **30** (Figure 12) has a violent inhibitory effect on EGFR^Del19/T790M/C797S^ and H1975 (EGFR^L858R/T790M/C797S^). In PC9 (EGFR^Del19/T790M/C797S^), the IC_50_ of compound **30** is 0.739 μM. **30** was well tolerated and there was no significant weight loss in the study. Compound **30** also significantly reduced the level of phosphorylated biomarker p-EGFR. In addition, **30** can optimise permeability by removing hydrogen bond donors and regulating compound PKA^63^. As a result, good pharmacokinetic characteristics were produced.

Ding and coworkers^64^ studied the antitumor activity of compound **31** (Figure 12) and confirmed that it is an effective inhibitor of EGFR^C797S^. Additionally, the weak inhibition of EGFR^WT^ by compound **31** helps to mitigate potential side effects, such as rashes or diarrhoea. However, the selectivity of compound **31** is suboptimal, as evidenced by a weight loss of approximately 21% after 14 days of treatment. This suggests that compound **31** may be potentially toxic, and its chemical structure requires further optimisation.

Kim and coworkers^65^ designed a series of EGFR inhibitors featuring 7H-pyrrolo [2,3-d] pyrimidine as the core structure. The optimal compound, designated as **32** (Figure 12), demonstrated activity against both EGFR^Del19/T790M/C797S^ and EGFR^L858R/T790M/C797S^ variants, exhibiting greater selectivity than the wild type. It exhibited strong inhibitory activity against both PC9 (EGFR^C797S^) and H1975 (EGFR^C797S^) cell lines, effectively inhibiting the phosphorylation of intracellular EGFR^Del19/T790M/C797S^ in a dose-dependent manner. The metabolite displayed moderate stability in human and mouse liver (24–57%) and was highly stable in human and mouse plasma. Pharmacokinetic data indicated that oral administration of compound **32** resulted in complete bioavailability and an acceptable pharmacokinetic profile, with a half-life of 2.3 h. The compound demonstrated favourable pharmacokinetic properties. *In vivo*, the tumour volume in the groups treated with oral compound **32** significantly decreased, with no observed weight loss or other clinical symptoms.

**Figure 13.**
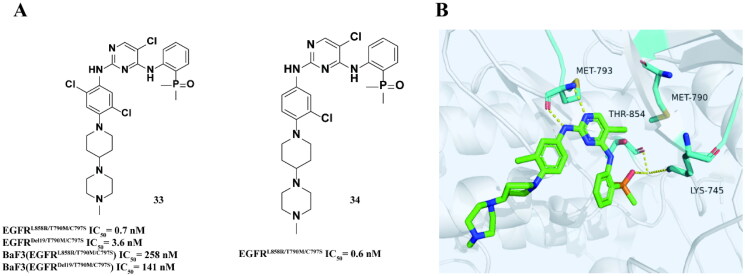
Chemical structures of brigatinib derivatives **33–34** and antitumor activity (A). Co-crystal structure of EGFR^T790M/C797S^ in complex with **33** (PBD: 7ER2) (B).

Ding and coworkers^66^ modified the 2-methoxy group of the core structure of brigatinib to avoid potential spatial conflicts and increase inhibitory activity. They found that compound **33** (Figure 13(A)) was more effective and had a stronger inhibitory effect on EGFR^TM1^ than **34**. Compound **34** (Figure 13(A)) can effectively inhibit EGFR^Del19/T790M/C797S^ and EGFR^L858R/T790M^, and the inhibitory effect on EGFR^WT^ is 94.1 times higher than that of **34**, indicating that **34** has high selectivity. In addition, **34** also showed inhibitory activity on the proliferation with EGFR^Del19/T790M/C797S^. Its oral bioavailability reached 81.7%. According to the eutectic structure of EGFR^T790M/C797S^ and **34** (Figure 13(B)), the hydrogen bond between 2-aminopyrimidine and MET793 formed in the hinge region, and the oxygen atom on the phosphine oxide group interacted with the carbonyl group of THR854 and the side chain NH_3_ of LYS745.

#### Hybrid derivatives of osimertinib and brigatinib

**Figure 14.**
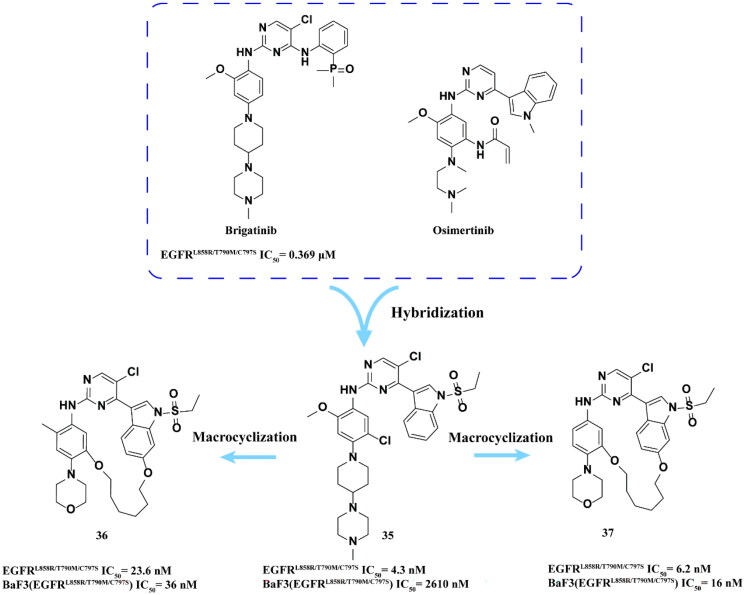
Chemical structures of osimertinib and brigatinib hybrid derivative **35–37** and antitumor activity.

Ding and coworkers^67^ designed and synthesised a series of conformational constrained 4-(1-ethylsufonyl-3-indolyl)-2-phenylaminopyrimidines derivatives. However, compound **35** (Figure 14) has very low inhibitory activity against BaF3 (EGFR^L858R/T790M/C797S^). To improve the cell activity compounds **36** and **37** (Figure 14) were obtained by macrocyclisation strategy. These two compounds have excellent inhibitory effects on EGFR^L858R/T790M/C797S^ and BaF3 (EGFR^L858R/T790M/C797S^). However, the oral PK properties of **37** are poor, which may limit its further development.

**Figure 15.**
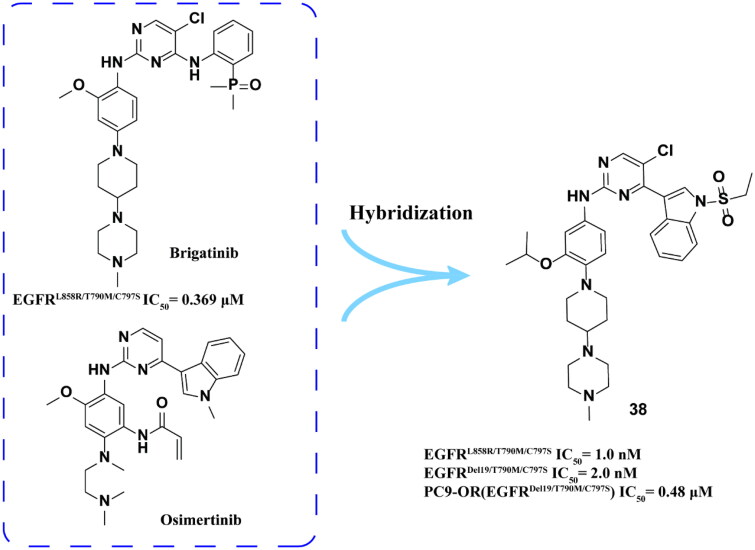
Chemical structure of osimertinib and brigatinib hybrid derivative **38** and antitumor activity.

Xie and coworkers^68^ hybridised osimertinib with brigatinib to synthesise a series of derivatives and finally obtained **38** (Figure 15) as a highly selective EGFR^T790M/C797S^ inhibitor. **38** showed good inhibitory activity against EGFR^L858R/T790M/C797S^, EGFR^Del19/T790M/C797S^ and EGFR^WT^. It also showed positive anti-proliferation ability in PC9-OR cells. In addition, **38** showed high selectivity and weak cytotoxicity after acting on human normal cell lines (MRC-9 and GSE-1 cells). In BaF3 and PC9-OR cells, **38** can effectively inhibit EGFR phosphorylation and induce apoptosis in a dose-dependent manner. In addition, it could significantly inhibit the growth of xenografted tumour in BaF3 (EGFR^Del19/T790M/C797S^) mice in a dose-dependent manner.

#### EAI001 derivatives

**Figure 16.**
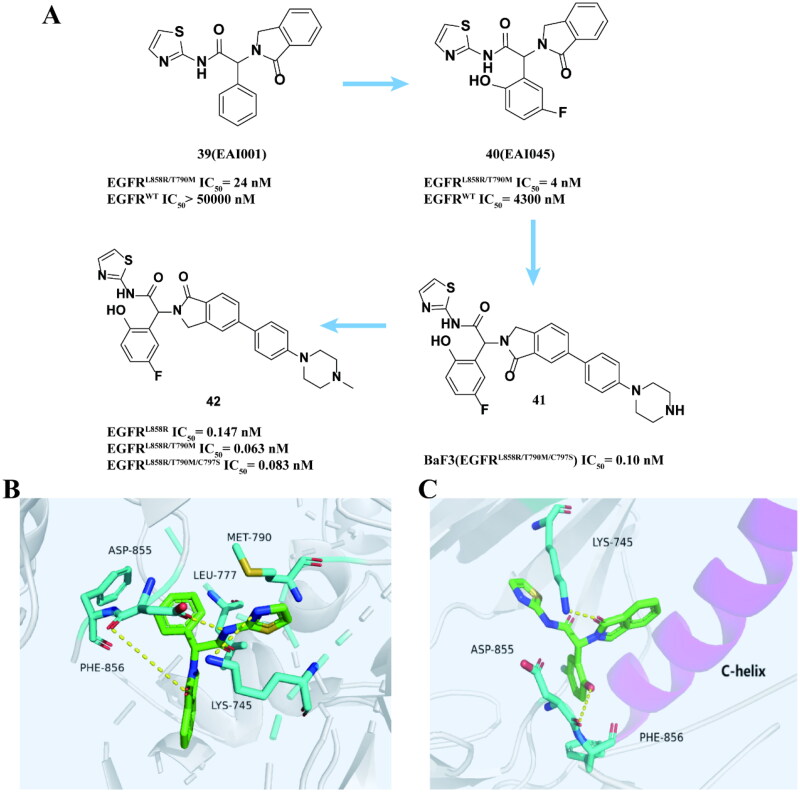
Chemical structures of **39 (EAI001)** derivatives **40–42** and antitumor activity (A). Co-crystal structure of EGFR^T790M/V948R^ in complex with **39** (PBD: 5D41) (B). Co-crystal structure of EGFR^T790M/C797S/V948R^ in complex with **40** (PBD ID: 5ZWJ) (C).

Thiazolamide **39 (EAI001)** (Figure 16) is a fourth-generation EGFR allosteric inhibitor developed by Eck and coworkers^69^ through high-throughput screening (HTS) of a chemical library comprising approximately 2.5 million molecules. It exhibits potent inhibitory activity against EGFR^L858R/T790M^, with an IC_50_ of 24 nM. It does not depend on C797S but instead binds to EGFR, which is located far from the ATP binding site. The crystal structure is shown in Figure 16(B). It indicates that the compound adopts a "Y" conformation within the allosteric pocket of EGFR^T790M/V948R^. Aminothiazole was partially positioned between the mutant janitor MET790 and the active site residue LYS745, forming a hydrogen bond with ASP855. Phenyl is situated in the hydrophobic gap at the rear of the pocket, where it interacts with LEU777 and PHE856^70^.

Further optimised the structure of **39** to give compound **40 (EAI045)** (Figure 16), which has a higher inhibitory activity to EGFR^L858R/T790M^ and stronger selectivity to EGFR^WT^ than compound **39**.^69^ Yun and coworkers^71^ showed the cocrystal structure of EGFR^T790M/C797S/V948R^ with compound **40** (Figure 16(C)). **40** binds to the activation domain like compound **40**. Additional hydrogen bonds between the carbonyl oxygen of 1-oxo isoindole and the LYS745 side chain were observed on the side chain of LYS745. Another additional hydrogen bond is formed between the hydroxyl group and PHE856. However, **40** not only partially inhibited EGFR phosphorylation in cells, but also had weak anti-proliferation activity against NSCLC containing L858R/T790M mutants. Studies have shown that **40** combined with cetuximab can prevent obvious tumour anti-proliferative response caused by EGFR dimerisation^72^. The same synergistic effect was observed in the C797S mutant cell line. **40** combined with cetuximab significantly reduced EGFR^L858R/T790M/C797S^ tumour growth.

However, this method is limited in clinical treatment because cetuximab can cause targeted wild-type EGFR-mediated toxicity. Janne and coworkers^73^ modified **40** to obtain a allosteric inhibitor **41** (Figure 16) with high efficiency and low toxicity. The functional groups of isoindole on carbon 6 showed good tolerance and produced stronger potency than **39**. Compound **41** has an inhibitory effect on carrying BaF3 (EGFR^L858R/T790M/C797S^). In mice carrying the L858R/T790M/C797S, **41** can effectively inhibit tumour growth. In addition, compared to using single drug, the combination of **41** and osimertinib can more effectively inhibit cell growth, increase cell apoptosis, and improve *in vitro* and *in vivo* therapeutic effects.

Although allosteric inhibitor **41** was found to be effective in *in vivo* and *in vitro* experimental models, it did not show the same efficacy in patient-derived cell lines or xenotransplantation models. Subsequently, the research team^74^ made modifications to compound **42**, resulting in the development of a new and more potent EGFR allosteric inhibitor **42** (Figure 16). It has strong inhibitory effect on EGFR^L858R^, EGFR^L858R/T790M^, EGFR^L858R/T790M/C797S^, and EGFR^LT/L747S^. It effectively reduces the phosphorylation of EGFR, Akt and ERK1/2. The higher intravenous clearance rate and bioavailability make **42** theoretically have better *in vivo* efficacy. In addition, it still has an inhibitory effect in combination with osimertinib. Therefore, it can be used as a single drug or in combination with EGFR-TKIs to treat EGFR mutant lung cancer. However, it is worth noting that there is currently no available data on the ability of compound **42** to penetrate the central nervous system, and its specificity towards the L747S mutation is higher compared to Osimertinib.

**Figure 17.**
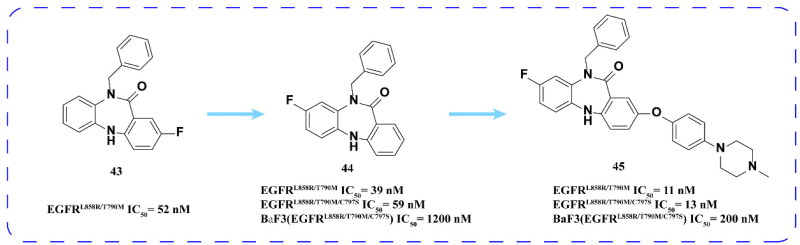
Chemical structures of **43** and its derivatives **44–45** and antitumor activity.

Gray and coworkers^75^ employed a comparable screening method to that of compound **39** and identified compound **43** as a potent inhibitor of EGFR^L858R/T790M^. Through fluorine transfer, compound **44** was derived from the structural optimisation of compound **43**, which led to the design and synthesis of a series of derivatives based on compound **44**. Among these derivatives, compound **45** exhibited the most significant inhibitory activity against EGFR^L858R/T790M/C797S^. However, similar to compound **40**, compound **45** did not affect the proliferation of BaF3 cell lines when administered alone. Notably, when combined with cetuximab, compound **45** demonstrated effective anti-proliferative activity against BaF3 (EGFR^L858R/T790M/C797S^) (Figure 17).

Based on the cyclisation strategy, Lee and coworkers^76^ partially replaced the 2-aminothiazole of compound **40** with quinazoline-4-one. Among the synthesised compounds, compound **46** (Figure 17) has good inhibitory activity against EGFR^L858R/T790M/C797S^.

**Figure 18.**
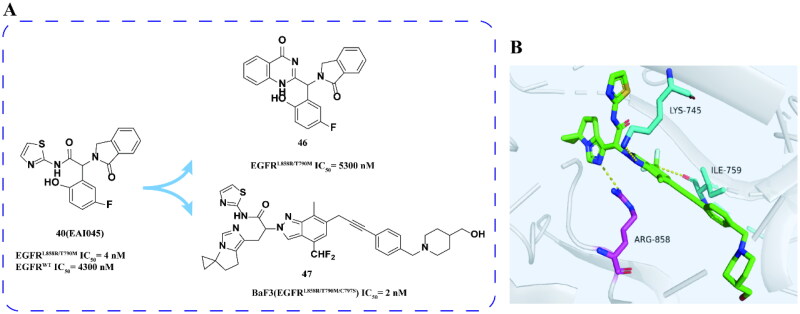
Chemical structures of **40 (EAI045)** derivatives **46–47** and antitumor activity (A). Co-crystal structure of EGFR^L858R/V948R^ in complex with **47** (PBD:8A2D) (B).

Jaeschke and coworkers^77^ optimised the structure of compound **40** to develop inhibitor **47** (Figure 18) with improved metabolic stability while maintaining good inhibitory activity on BaF3 (EGFR^L858R/T790M/C797S^). Compound **47** displayed minimal sensitivity in most cells (IC_50_ > 3 μM) but significantly inhibited tumour growth in the BaF3 (EGFR^L858R/C797S^) allotransplantation model, achieving an 84% tumour regression rate. Notably, tumour regression continued to improve after 20 days, with 70% of mice showing no measurable tumours and exhibiting good tolerance. The crystal structure analysis (Figure 18(B)) revealed that the carbonyl group of **47**’s isoindolinone core forms a hydrogen bond with LYS745. The high selectivity of compound **47** can be attributed to the direct interaction between its imidazole moiety and ASP858. Furthermore, compound **47** demonstrated wide tissue distribution, low total plasma clearance, moderate half-life, and 22% oral bioavailability. With promising therapeutic potential for EGFR^L85858R/T790M/C797S^, compound **47** exhibits good drug efficacy and competitive pharmacokinetic properties, making it a viable candidate for further drug development.

**Figure 19.**
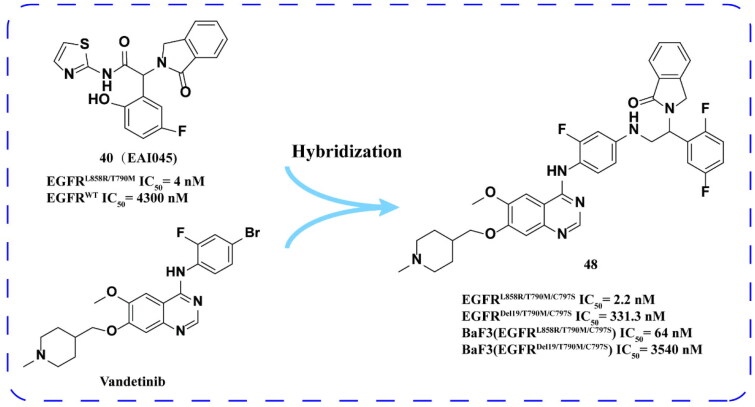
Chemical structure of **40 (EAI045)–vandertinib** hybridised derivative **48** and antitumor activity.

When Xu and coworkers^78^ coupled allosteric inhibitor **40 (EAI045)** with ATP competitive inhibitor vandetanib, they found that the most effective inhibitor **48** (Figure 19) had an IC_50_ of 2.2 nm for EGFR^L858R/T790M/C797S^. This compound effectively inhibits the proliferation of BaF3 (EGFR^L858R/T790M/C797S^) cells. However, compound **48** exhibited high clearance rate and only had an oral bioavailability of 0.55%. Furthermore, its inhibitory activity against EGFR^Del19/T790M/C797S^ was moderate, in line with its kinase activity, but its efficacy against BaF3 (EGFR^Del19/T790M/C797S^) cell line was low.

**Figure 20.**
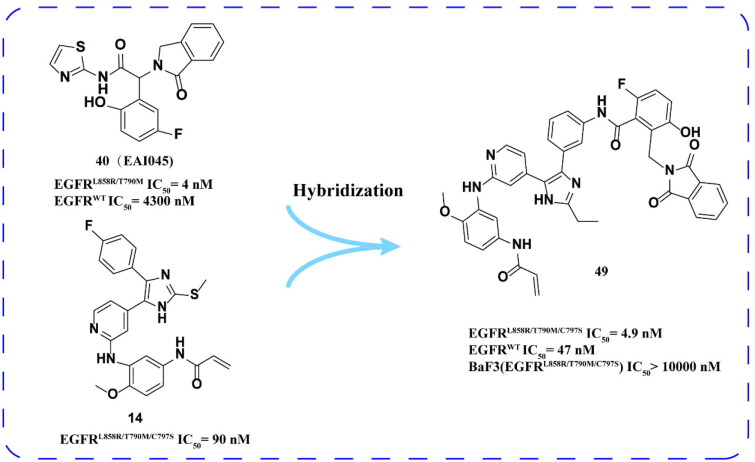
Chemical structure and antitumor activity of **40 (EAI045)–14** hybrid derivative **49**.

Laufer and coworkers^79^ designed and synthesised compound **49** (Figure 20) based on ATP competitive inhibitor **14** and allosteric inhibitor **40 (EAI045)**. Compound **49** has low inhibitory activity to EGFR^L858R/T790M/C797S^ and is not sensitive to BaF3 (EGFR^L858R/T790M/C797S^).

**Figure 21.**
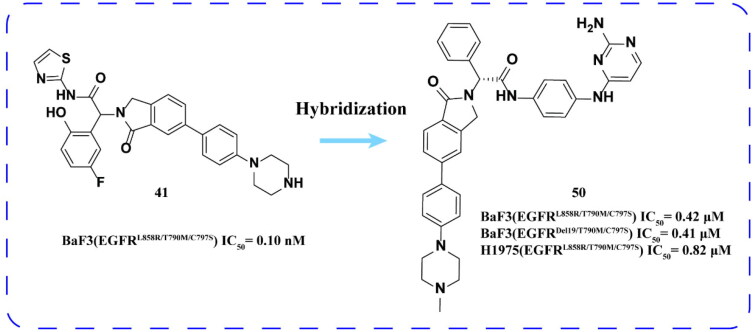
Chemical structure of compound 50 and antitumor activity.

Qi and coworkers^80^ designed and synthesised a series of novel EGFR inhibitors based on the binding mode of the EGFR inhibitor JBJ-04-125-02. The evaluation of biological activity revealed that the optimal compound **50** (Figure 21) exhibited significant inhibitory activity against BaF3 (EGFR^L858R/T790M/C797S^) and BaF3 (EGFR^Del19/T790M/C797S^) cell lines. Notably, it demonstrated superior inhibitory effects on the mutant NSCLC cell line NCI-H1975 (EGFR^L858R/T790M/C797S^) compared to osimertinib and JBJ-04-125-02.

#### JND3229 derivatives

**Figure 22.**
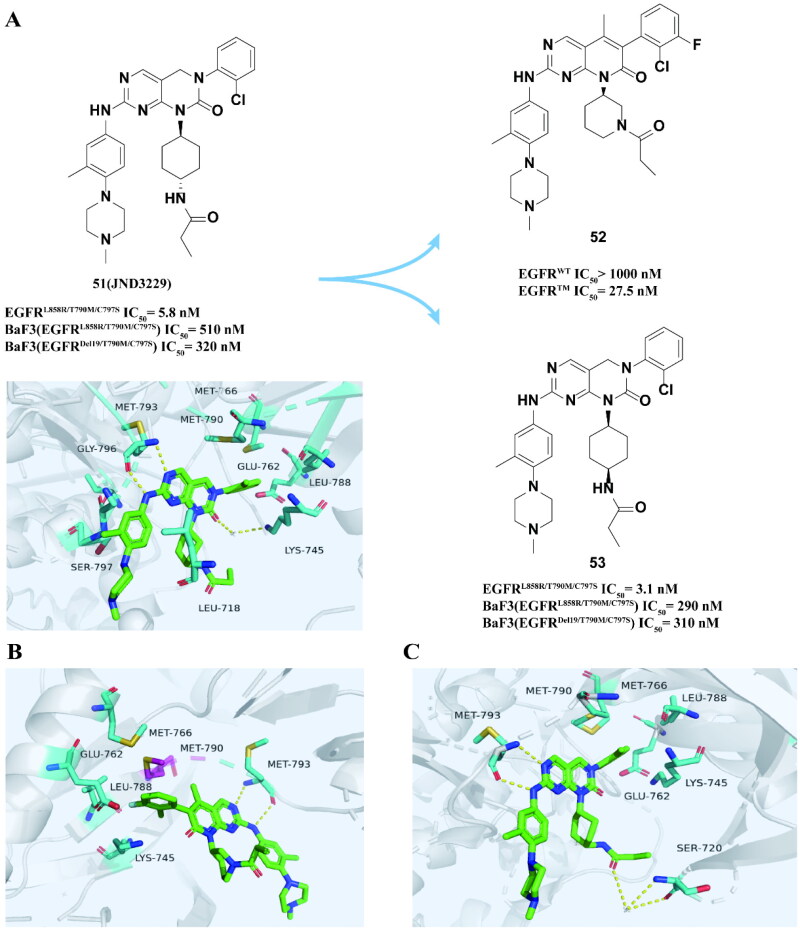
Chemical structures of **51 (JND3229)** and its derivatives **52–53** and antitumor activity. Co-crystal structure of EGFR^L858R/T790M/C797S^ in complex with **51** (PBD: 5ZTO) (A). Co-crystal structure of EGFR^T790M/C797S^ in complex with **52** (PBD: 6JRJ) (B). Co-crystal structure of EGFR^T790M/C797S^ in complex with **53** (PBD: 6JRX) (C).

Ding and coworkers^81^ randomly screened 3000 compounds from the kinase inhibitor library to obtain a compound **51 (JND3229)** (Figure 22) with an IC_50_ of 5.8 nM for EGFR^L858R/T790M/C797S^. In addition, the compound can effectively inhibit the proliferation of BaF3 (EGFR^L858R/T790M/C797S^) and BaF3 (EGFR^Del19/T790M/C797S^) and has antitumor activity in the transplanted tumour model of BaF3 (EGFR^Del19/T790M/C797S^) cell line mice, which is better than that of **51** combined with cetuximab. However, the selectivity to wild-type cells is low. From the eutectic structure of EGFR^L858R/T790M/C797S^ and **51** (Figure 22(A)), MET793 forms a bidentate hydrogen bond with 7-amino-3-pyrimidine-4-dihydropyrimidine-2-one, 2-chlorophenyl towards the back hydrophobic pocket. In addition, the carbonyl group on the pyrimidine-2-one nucleus forms a hydrogen bond with LYS745 under the action of water molecules. Another hydrogen bond mediated by water molecule is formed between the LEU718 and NH groups on the propylamine group.

The team^82^ designed and synthesised a series of 5-methylpyrimidinopyridone compounds based on **51**. The optimal compound **52** (Figure 22) has an IC_50_ of 27.5 nM to EGFR^L858R/T790M/C797S^ and has almost no inhibitory effect on EGFR^WT^. In addition, the compound has moderate water solubility and cell permeability. The eutectic structure of EGFR^T790M/C797S^ and **52** shows (Figure 22(B)) that the 7-oxo [2,3-d] pyrimidine nucleus forms a typical bidentate hydrogen bond with MET793. 2-Chloro-3-fluorophenyl goes directly into the back hydrophobic pocket. It is worth noting that 5-methyl interacts with MET790 through hydrophobic interaction, which contributes to high selectivity for EGFR with T790M mutation^82^. This study overcomes the disadvantage of poor selectivity of **52** pairs of wild-type cells and provides a theoretical basis for further pharmacokinetic optimisation of pyrimidine ketone EGFR inhibitors.

Lu and coworkers^83^ also designed a range of new reversible 2-oxo-3,4-dihydropyrimido[4, 5-d] Pyrimidines on the **53** structure as EGFR^C797S^ inhibitors. The binding mode of **53** is like that of **51**, but the hydrogen bond between the carbonyl group of the propionamide group and SER720 is mediated by the water molecule. **53** (Figure 22) effectively inhibited EGFR^L858R/T790M/C797S^ and inhibited the proliferation of BaF3 (EGFR^L858R/T790M/C797S^) and BaF3 (EGFR^Del19/T790M/C797S^). In addition, the compound inhibited the phosphorylation of BaF3 (EGFR^L858R/T790M/C797S^) and BaF3 (EGFR^Del19/T790M/C797S^) in a dose-dependent manner. The crystal structure shows that **53** (Figure 22(C)) bound to EGFR^T790M/C797S^ with a reversible “U-shaped” conation with the “hinge” residue MET793. The 2-chlorophenyl group was directed towards the hydrophobic back pocket composed by LYS745, GLU762, LEU788, MET766, and MET790. Overall, compound **53** demonstrated enhanced inhibitory activity compared to **51**.

#### 9-cyclopentyl-N^2^-(4-(4-methylpiperazin-1-yl) phenyl)-N^8^-phenyl-9H-purine-2,8-diamine derivatives

Yang and coworkers^84^ synthesised a series of novel N^2^-(4-(4-methylpiperazin-1-yl)phenyl)-N^8^-phenyl-9H-purine-2,8-diamine derivatives. The optimal compound **54** (Figure 22) is an effective inhibitor of EGFR^WT^. This compound exhibited high selectivity towards kinase spectrum, showing minimal inhibitory activity across the 20 protein kinases tested. In EGFR overexpressed cell lines Calu-3, H292, and A431, compound **54** demonstrated superior inhibitory effects compared to gefitinib, although it had no impact on EGFR^C797S^. It was noted that excessive CGRP levels hindered the development of compound **54** as a potential candidate.

**Figure 23.**
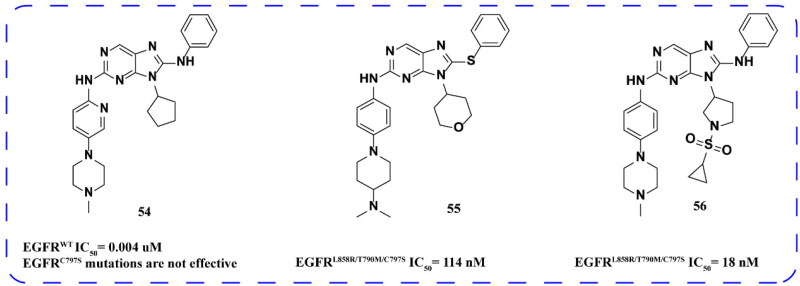
Chemical structures of **54** and its derivatives **55–56** and antitumor activity.

Zhang and coworkers^85^ synthesised and evaluated 2,9-disubstituted 8-phenylthio/phenylsulfinyl-9H-purine compounds as new EGFR inhibitors. The optimal compound **55** (Figure 23) demonstrated moderate inhibitory activity against EGFR^L858R/T790M/C797S^.

The team^86^ also incorporated sulphonyl heterocyclic substituents into compound **56** to develop a series of novel 9-heterocyclic substituted 9H-purine derivatives. Among these derivatives, compound **56** exhibited the best activity with an IC_50_ of 18 nM against EGFR^L858R/T790M/C797S^ (Figure 23). Furthermore, compound **56** showed significant inhibition of EGFR phosphorylation, induction of apoptosis, cell cycle arrest in G0/G1 phase, and inhibition of colony formation in HCC827 cell line in a concentration-dependent manner.

#### Angew2017-7634-1 and its derivatives

**Figure 24.**
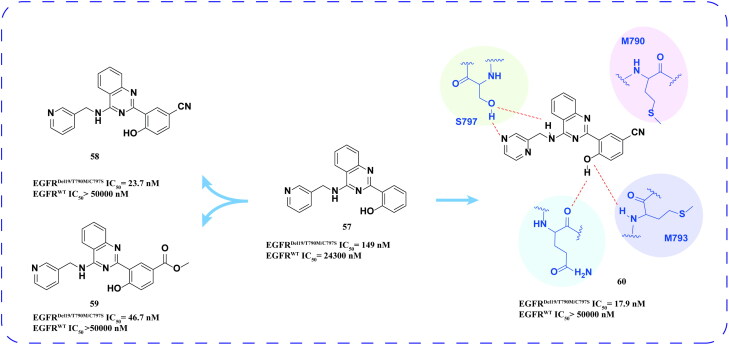
Chemical structures of **57** and its derivatives **58–60** and antitumor activity.

Hong and coworkers^87^ identified compound **57** (Figure 24) as the lead compound from a pool of approximately 330 000 drug candidates, with an IC_50_ value of 149 nM for EGFR^Del19/T790M/C797S^. Following the optimisation of the phenyl and pyridine groups of compound **57**, compounds **58**, **59**, and **60** were synthesised, which effectively inhibited EGFR^Del19/T790M/C797S^ (Figure 24). Furthermore, these compounds exhibited high selectivity for EGFR^WT^. Molecular docking studies revealed that the hydroxyl groups on the benzene ring formed two hydrogen bonds with GLU791 and MET793 in the hinge region of EGFR^Del19/T790M/C797S^, while a hydrophobic interaction occurred between the 2-benzene ring of compound **60** and MET790. Additionally, the formation of an extra hydrogen bond between the (pyrazine-2-yl)methylamino group and SER797 significantly contributes to the high potency of compound **60**.

**Figure 25.**
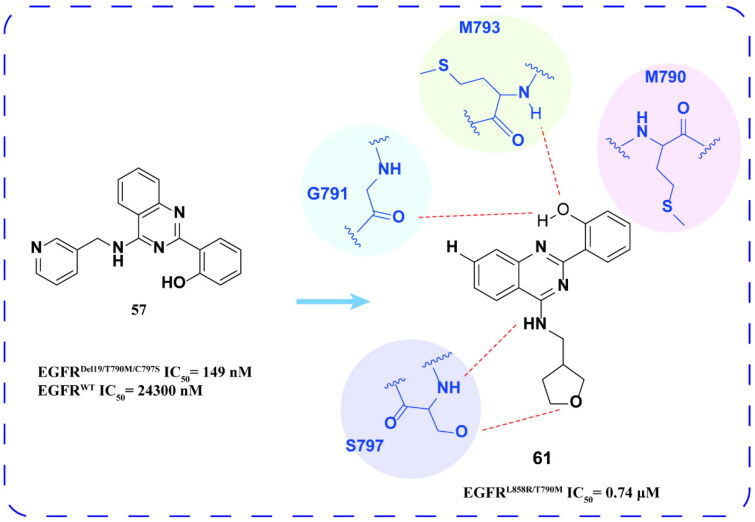
Chemical structures of **57** and its derivatives **61** and antitumor activity.

Zhu and coworkers^88^ optimised the N-9 (H), C-2 (aniline group), and C-8 (aniline group) positions of the purine backbone of the lead compound **57** to obtain the optimal compound **61**. The compound **61** (Figure 25) showed intense inhibitory activity against EGFR^L858R/T790M^ double mutation and good inhibitory activity against A549, NCI-H460, and H1975 cells. At the same time, compound **61** induced late apoptosis of A549 cells in a concentration-dependent manner. In the HCC827 and H1975 transplanted tumour models, compound **61** could produce a significant antitumor effect. Docking data revealed that the phenol substitution at position 2 of quinazoline was oriented towards M790 and protruded into the hydrophobic cavity. It forms hydrogen bonds with the hinge region of M793 and with G791. The side chain amino group substitution at position 4 extended towards the solvent region and formed two hydrogen bonds with the amino acid residue S797. In addition, the presence of a significant cavity in the para position of phenol suggests potential for new design strategies in future structural modifications.

**Figure 26.**
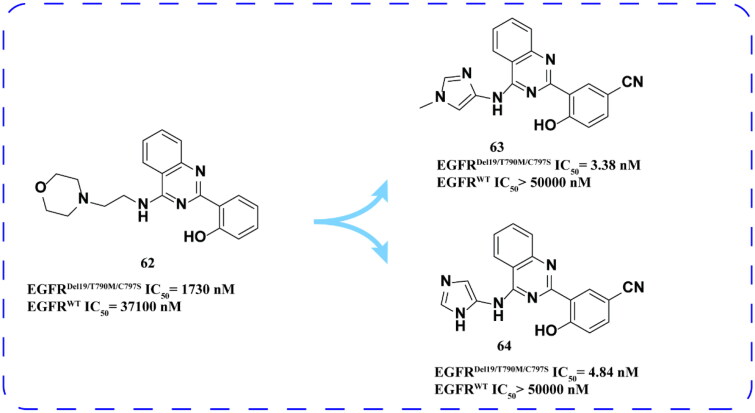
Chemical structures of **62** and its derivatives **63–64** and antitumor activity.

Hong and coworkers^89^ continued to screen another chemical library containing about 370 000 drug molecules. Compound **62** (Figure 26) was identified to have low inhibitory activity against EGFR^Del19/T790M/C797S^ and was used as a lead compound to synthesise a series of derivatives. Among them, compounds **63** and **64** (Figure 26) have inhibitory activity and good selectivity to EGFR^Del19/T790M/C797S^.

#### Other structural compounds

Furthermore, it is essential to employ efficient, rapid, and cost-effective HTS methods to systematically screen and investigate novel structures targeting EGFR^C797S^. This approach will facilitate large-scale targeted screening of drugs within existing chemical libraries.

**Figure 27.**
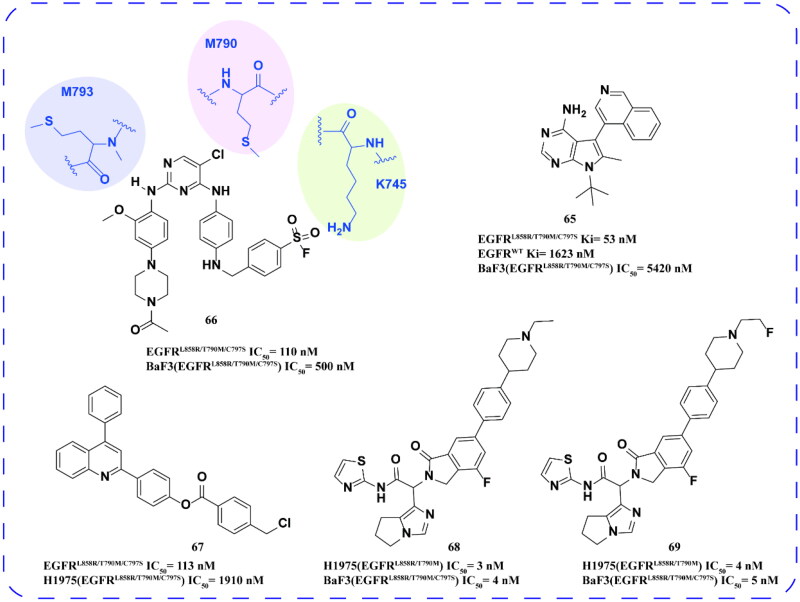
Chemical structures of compounds **65–69** and antitumor activity.

Gustafson and coworkers^90^ showed that atropine isomer **65** (Figure 27) is also an effective EGFR inhibitor with strong inhibitory activity on EGFR^L858R/T790M/C797S^ and weak inhibitory activity on EGFR^WT^. And **65** had moderate inhibitory activity on BaF3 (EGFR^L858R/T790M/C797S^) cells. Therefore, atropine isomerisation also plays an important role in the development of EGFR inhibitors.

Mor and coworkers^91^ synthesised a series of sulphonyl compounds by adding functional groups that can covalently bind with the electrophilic portion of LYS745. Among them, the best compound **66** (Figure 27) has inhibitory activity on EGFR^L858R/T790M/C797S^, in addition, it can also inhibit the proliferation of the BaF3 (EGFR^L858R/T790M/C797S^) cell line. The covalent binding of compound **66** to LYS745 can be verified by LCHRMS analysis. Thus, it provides an idea for finding covalent EGFR-TKI combined with LYS745.

Wackett et al. designed and synthesised a series of quinoline derivatives, the optimal compound **67** can effectively inhibit EGFR^L858R/T790M/C797S^. It showed good inhibitory activity on the H1975 (EGFR^L858R/T790M/C797S^). Falk et al.^92^ designed and synthesised a series of isoindole compounds as EGFR-TKIs. Compounds **68** and **69** significantly inhibited the proliferation of H1975 (EGFR^L858R/T790M^) and BaF3 (EGFR^L858R/T790M/C797S^) (Figure 27).

**Figure 28.**
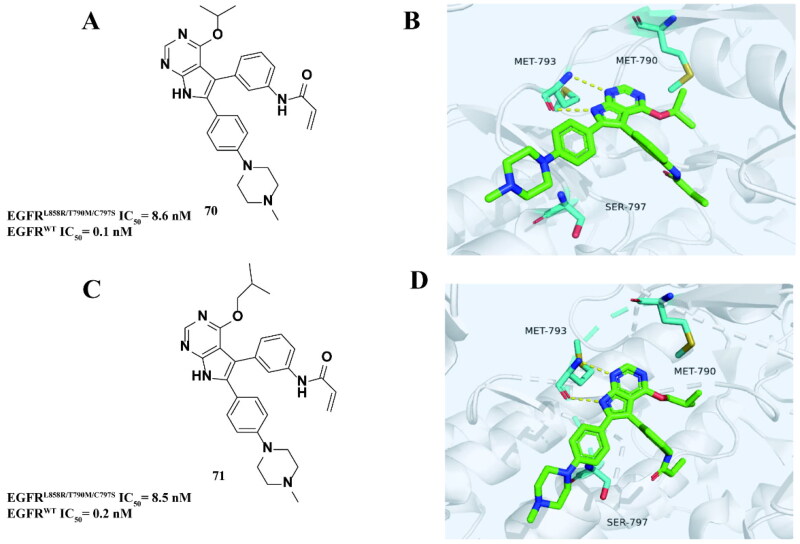
Chemical structure of compounds **70** and antitumor activity (A). Co-crystal structure of EGFR^T790M/C797S^ in complex with **70** (PBD: 6S89) (B). Chemical structure of compounds **71** and antitumor activity (C). Co-crystal structure of EGFR^T790M/C797S^ in complex with **71** (PBD: 6S8A) (D).

Rauh and coworkers^93^ established a fast-advancing Mitsunobu reaction program for the synthesis of 3-substituted pyrrolopyrimidin-4-ones and 4-substituted pyrrolopyrimidines. Among the synthesised compounds, **70** (Figure 28(A)) and **71** (Figure 28(C)) had violent inhibitory effects on EGFR^L858R/T790M/C797S^. The eutectic structure of EGFR^T790M/C797S^ with **70** (Figure 28(B)) and **71** (Figure 28(D)) shows that the pyrrolidine scaffold is bound to MET793 by a bidentate hydrogen bond. In addition, isopropoxy and isobutoxy interact with MET790 with a large surface area, which may be the reason for the high potency of these compounds.

**Figure 29.**
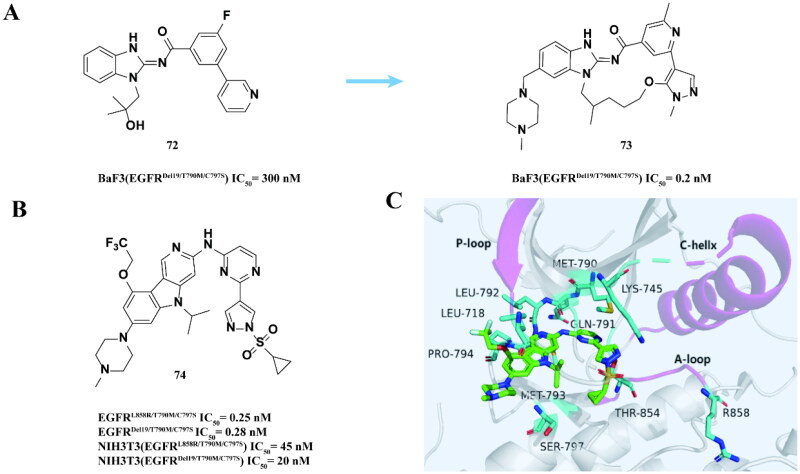
Chemical structures of compound **72** and its derivative **73** and antitumor activity (A). Chemical structure of **74** and antitumor activity (B). Crystal structure of **74** (PBD: 6LUB) (C).

McConnell and coworkers^94^ screened and optimised a highly selective aminobenzimidazole compound **72** from the kinase inhibitor library as an effective inhibitor for EGFR^Del19/T790M/C797S^. To improve the activity, a highly selective inhibitor **73** was obtained after macrocyclisation (Figure 29(A)). The IC_50_ to BaF3 (EGFR^Del19/T790M/C797S^) is 0.20 nM. In the PC9 (EGFR^Del19/T790M/C797S^) xenograft tumour model mice, after treatment, the tumour regressed significantly in group **73**, and the tumour inhibition rate (TGI = 121%) was higher than that in the control group. The strategy of improving the potency of the compound by limiting the conformational space of the ligand to "active" conformation through macrocyclic interaction is a key factor for success.

Sakamoto and coworkers^95^ conducted HTS of EGFR^Del19/T790M/C797S^ inhibitors from a chemical library of over 1 million compounds. They discovered that compound **74** (Figure 29(B)) exhibited sensitivity to EGFR^L858R/T790M/C797S^ and EGFR^Del19/T790M/C797S^, with IC_50_ values of less than 1 nM, as well as to NIH3T3 cell lines containing the triple mutant EGFR. In mouse models, the compound demonstrated inhibitory effects on transplanted tumours of NIH3T3 (EGFR^Del19/T790M/C797S^) cell lines at a dose of 100 mg/kg. Crystal structure analysis revealed that **74** (Figure 29(C)) functions as a non-covalent ATP competitive inhibitor of EGFR^Del19/T790M/C797S^. This compound achieves effective inhibitory activity and mutation selectivity through multiple interactions with the **α** C-helix-in conformation of EGFR.

**Figure 30.**
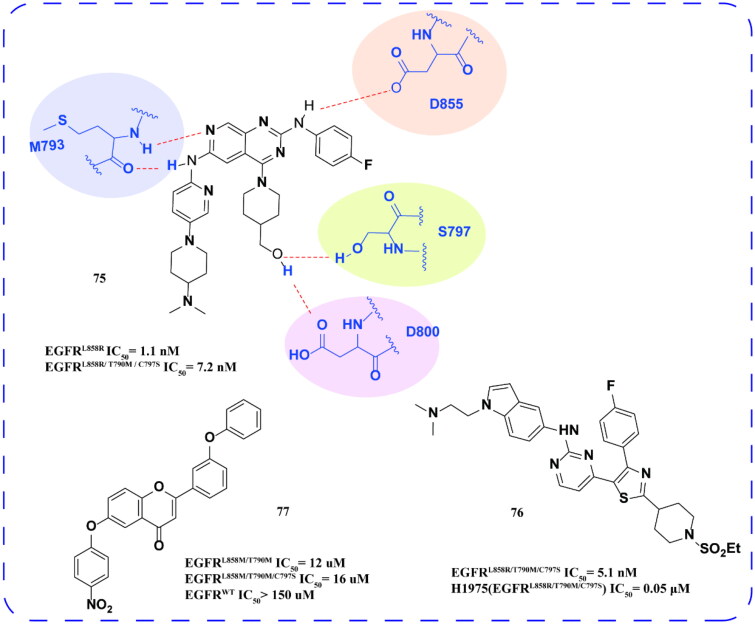
Chemical structures of **75–77** and antitumor activity.

Zhang and coworkers^96^ designed and synthesised a series of 2,4,6-trisubstituted pyrido[3,4-d]pyrimidine derivatives. Among them, the optimal compound **75** (Figure 30) can inhibit both EGFR^L858R^ and EGFR^L858R/T790M/C797S^, and inhibit the growth of HCC827 and H1975 cells. Docking shows that the key hydrogen bond is formed between compounds **75** and M793. 4-Fluorophenyl points to the back hydrophobic pocket. In addition, 4-fluorophenyl and piperidine-4-methanol have hydrogen bonding with D855, D800, and S797, respectively.

Rho and coworkers^97^ designed compound **OBX02-011** and tested its *in vitro* and *in vivo* therapeutic effects. The compound has a strong inhibitory effect on EGFR^Del19/T790M/C797S^ and EGFR^L858R/T790M/C797S^, but a weak inhibitory effect on EGFR^WT^. In BaF3 cells, it has a strong inhibitory effect on EGFR triple mutation. **OBX02-011** treatment inhibited the activity of EGFR in the triple mutation of EGFR and the activity of downstream signal proteins Akt and Erk. *In vivo*, **OBX02-011** induced a significant decrease in tumour growth in mice with BaF3EGFR triple mutation in a dose-dependent manner, and there were no obvious signs of weight loss and toxicity in mice. The compound is currently under clinical development and clinical trials will start soon, but the molecular structure has not been disclosed.

Yang and coworkers^98^ used HTS to screen compounds targeting C797S mutations. The optimal compound **77** (Figure 30) can effectively inhibit EGFR^L858R/T790M^, EGFR^L858R/T790M/C797S^, and EGFR^Del19/T790M/C797S^. It can significantly stagnate cells in the G0/G1 phase and promote apoptosis in a concentration-dependent manner. In addition, **77** significantly inhibited the phosphorylation of EGFR and the activation of downstream signal transduction proteins AKT and ERK. The tumour growth inhibition (TGI) rate of the **77** high-dose group was 62.9%, which was stronger than that of the brigatinib group. The tumour tissues were stained with Ki67 and HE, and the results showed that the cell proliferation in the treatment group was significantly weaker than that in the control group, and there was no obvious damage to the main organs of mice after administration. Compound **77** has good pharmacokinetic characteristics, and the oral bioavailability is 30.72%. Overall, compound **77** was demonstrated to inhibit tumour growth *in vivo* safely and effectively.

Giovanna and coworkers^99^ obtained nitro derivative **76** (Figure 30) through HTS based on flavonoid structure. It has a good inhibition effect on EGFR^L858M/T790M^ and EGFR^L858M/T790M/C797S^ and good selectivity.

### Anti-C797S fourth-generation EGFR-TKIs which have entered the clinical stage

Currently, there are no approved marketed drugs for the treatment of EGFRC797S. However, according to the data provided by the smart bud new drug information database, a variety of fourth generation EGFR targeted drugs targeting drug resistance mutations have entered the clinical trial stage, as shown in Table 1. This part of data is from https://mpharmsnap.zhihuiya.com.

**Table 1.** The fourth generation of EGFR-TKIs has entered the clinical trial stage.

Drugs	Company	Stage	Reference
JIN-A02	J INTS BIO	Phase 1/2	^100^
BLU-945	Blueprint Medicines Corp. (Cambridge, MA)	Phase 1/2 (suspend or terminate)	^101^
BBT-176	Bridge Biotherapeutics, Inc. (Seongnam, South Korea)	Phase 1/2 (suspend or terminate)	^102^
BBT-207	Bridge Biotherapeutics, Inc. (Seongnam, South Korea)	Phase 1/2	^103^
BPI-361175	Betta Pharmaceuticals Co., Ltd. (Hangzhou, China)	Phase 1/2	^104^
TAS-3351	Taiho Pharmaceutical Co., Ltd. (Chiyoda, Japan)	Phase 1/2	^105^
H002	Nanjing Hongyun Biotechnology Co., Ltd. (Nanjing, China)	Phase 1/2	^106^
TQB3804	Chia Tai Tianqing Pharmaceutical Group Co., Ltd. (Lianyungang, China)	Phase 1/2	^107^
BDTX-1535	Black Diamond Therapeutics (Cambridge, MA)	Phase 1/2	^108^
PH009-1	Suzhou Puhe Medical Technology Co., Ltd. (Suzhou, China)	Phase 1/2	^109^
QLH11811	Qilu Pharmaceutical Co., Ltd. (Jinan, China)	Phase 1	^110^
ES-072	Zhejiang Bosheng Pharmaceutical Co., Ltd. (Zhejiang, China)	Phase 1	^111^
BLU-701	Zai Lab (Shanghai) Co., Ltd. (Shanghai, China)	Phase 1	^112^
BAY 2927088	Bayer (Leverkusen, Germany)	Phase 1	^113^
VRN-11	Voronoi, Inc. (Incheon, South Korea)	Phase 1	^92^

The Korean company J INTS BIO has developed a novel oral EGFR-TKI, designated **JIN-A02**, which exhibits a significantly enhanced inhibitory effect on the growth of EGFR^Del19/T790M/C797S^ cells, with an IC_50_ of 92.1 nM.^114^ In the PDC model, dosages of 50 mg/kg and 60 mg/kg administered once daily (QD) significantly inhibited tumour growth in mice, achieving TGI rates of 91.7% and 95.7%, respectively. The initial human dose exploration conducted in a phase 1 clinical study demonstrated a reduction in tumours and brain metastases, with no reported cases of rash, diarrhoea, or cardiac toxicity. At present, the safety, tolerability, pharmacokinetics, and anti-tumour activity of NCT05394831 in patients with EGFR-mutated advanced NSCLC are being evaluated in phase 2 clinical trials, and the results have not yet been announced.^115^

Lee and coworkers^116^ developed a reversible ATP competitive inhibitor **BBT-176** (Figure 29) against EGFR^C797S^. It has a significant inhibitory effect on EGFR^Del19/T790M/C797S^ and EGFR^L858R/C797S^. It can also effectively inhibit BaF3EGFR^Del19/T790M/C797S^ and BaF3 (EGFR^L858R/T790M/C797S^). In the mouse models, **BBT-176** violently inhibited tumour growth and induced tumour regression.^117^ However, the results announced by Bridge Biotherapeutics (Seongnam, South Korea) at the WCLC conference in 2023 were disappointing, with only one out of 18 patients receiving treatment achieving a partial response (PR). As a result, the trial has been suspended or terminated.

**BPI-361175** is a novel, potent, and selective oral small-molecule EGFR inhibitor that has been independently developed by Betta Pharmaceuticals (Hangzhou, China). It is designed to treat solid tumours, including advanced NSCLC with EGFR^C797S^ and other EGFR-related mutations. According to previously disclosed preclinical data, **BPI-361175** exhibits inhibitory activities of 15 nM and 34 nM against EGFR^Del19/T790M/C797S^ and EGFR^L858R/T790M/C797S^, respectively, demonstrating high selectivity.^104^ The current phase 2 clinical trial (NCT05393466) aims to evaluate the safety, tolerability, pharmacokinetics, and anti-tumour activity of **BPI-361175** in advanced NSCLC patients with EGFR^C797S^ mutation and other EGFR related mutations.

**ES-072** (Figure 29) is an irreversible selective EGFR covalent 4th generation EGFR-TKIs independently developed by Zhejiang Bosheng Pharmaceutical Co., Ltd. (Zhejiang, China). Its IC_50_ to H1975 (EGFR^T790M/L858R/C797S^) is 1.06 μM. Zhou and coworkers^111^ completed the phase I human clinical trial (CTR20180074) for the first time in patients with NSCLC with T790M mutation. The results show that **ES-072** is safe and well tolerated in patients with NSCLC. Compared with the first-generation of EGFR-TKIs, the incidence of rash and diarrhoea after **ES-072** treatment was 15.8% and 10.5%, respectively, indicating that **ES-072** was more selective to mutant EGFR. However, **ES-072** has cardiotoxicity, showing a high incidence (57.9%) of prolonged QT interval, so the monitoring of patients’ cardiac function indicators can be strengthened in follow-up clinical trials.

**BLU-945** (Figure 29) was developed by Blueprint Medicines (Cambridge, MA) to target EGFR^T790M/C797S^ and other EGFR^T790M^ resistance mutations secondary to osimertinib resistance. Dineen and coworkers^118^ found that **BLU-945** is significantly effective against BaF3 (EGFR^L858R/T790M/C797S^) and BaF3 (EGFR^Del19/T790M/C797S^). In BaF3 (EGFR^L858R/T790M/C797S^) and BaF3 (EGFR^Del19/T790M/C797S^) mutant mice, BLU-945 has a strong tumour inhibitory effect. The results of the phase 2 clinical study (CTR20221771) indicated that in the monotherapy group, higher doses of **BLU-945** demonstrated enhanced anti-tumour activity, with two patients confirmed to have achieved PR. In the group receiving treatment with **BLU-945** combined with osimertinib, a greater number of patients were observed to experience tumour shrinkage. During the continuous dose escalation process, 10 patients achieved a PR, of which four have been confirmed. However, there were 12 cases of dose-limiting toxicity among the patients in the high-dose group, primarily attributed to liver dysfunction. The effective dose of the combination therapy group exceeded the threshold for limiting toxicity, thereby restricting its clinical application. The trial has currently been suspended or terminated^119^^,^^120^.

The preclinical results of **TQB3804** (Figure 29) developed by Zhengda Tianqing (Lianyungang City, China) showed that its IC_50_ for EGFR^WT^ and EGFR^L858R/T790M/C797S^ was 7.92 and 0.218. In the BaF3 (EGFR^Del746-750/T790M/C797S^) xenotransplantation model, **TQB3804** inhibits the growth of the transplanted tumour model by inhibiting phosphorylated EGFR (p-EGFR), phosphorylated AKT (p-AKT), and phosphorylated extracellular signal-regulated kinase (p-ERK) targeting EGFR tertiary mutation.^107^ The phase I clinical trial (NCT04128085) of the drug was launched in November 2019. However, there are no reports on the safety and remission rate and control rate of C797S mutant drug-resistant cancer patients^107^.

**BDTX-1535** is a fourth-generation irreversible covalent inhibitor of the EGFR developed by Black Diamond Therapeutics (Cambridge, MA)^121^. Additionally, **BDTX-1535** demonstrates favourable brain penetration properties. The 2024 AACR conference reported efficacy data indicating that six out of 11 patients who were resistant to osimertinib and treated with BDTX-1535 achieved a PR (five complete responses, one unconfirmed PR), resulting in an ORR of 55%. Low doses of **BDTX-1535** (15–200 mg) administered once daily did not exhibit dose-limiting toxicity. However, patients receiving higher doses (300–400 mg) once daily experienced varying degrees of dose-limiting toxicity.

## Summary and prospect

Numerous fourth-generation inhibitors targeting EGFR^C797S^ have demonstrated effectiveness in preclinical studies. Several EGFR inhibitors currently progressing through phase I/II clinical trials have also shown efficacy in human patients. These results suggest that the development of fourth-generation inhibitors holds promise for overcoming resistance to third-generation EGFR inhibitors. Despite these advancements, substantial limitations and challenges persist^25^.

**Figure 31.**
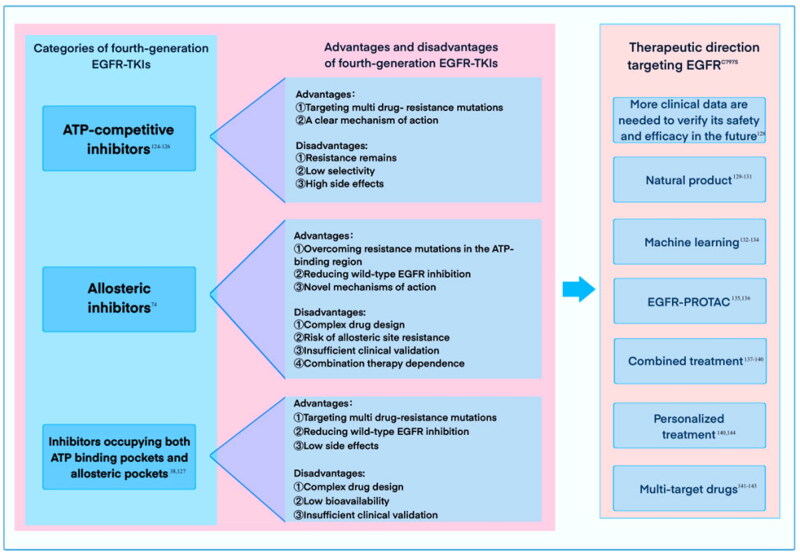
Categories, advantages, and disadvantages of fourth-generation EGFR-TKIs and new therapeutic direction targeting EGFR^C797S^.

Current fourth-generation EGFR-TKI structures are mostly ATP-competitive inhibitors, often forming hydrogen bonds with the hinge region of the kinase domain to mimic the ATP binding pattern^122^. Therefore, high concentrations of ATP may reduce its efficacy. For example, trisulfide substituted imidazole, 4-aminopyrazole, and pyrrolo[2,3-d]pyrimidine, which typically have significant activity against EGFR triple mutations, including C797S, but have low selectivity; 2-aryl-4-aminoquinazoline derivatives exhibit high selectivity for EGFR^Del19/L858R/T790M^ and exhibit inhibitory activity but poor cellular activity^123^. 2-Aniline-8,9-disubstituted-9H-purine and 2,4,6-trisubstituted pyrimidine[3,4-d]pyrimidine are effective against EGFR^L858R/T790M/C797S^, but their activity *in vitro* and *in vivo* has not been further evaluated. In addition, most ATP-competitive inhibitors are often used with rash and diarrhea^124^. Allosteric inhibitors that bind to EGFR allosteric pockets have some drawbacks, although they exhibit high target selectivity with low *in vivo* side effects. For example, the allosteric inhibitor EAI045 and its derivatives have reduced inhibition of EGFR^Del19/T790M/C797S^ due to limited allosteric pocket space, while inhibiting EGFR^L858R/T790M/C797S^ mutations. In addition, the results of EAI045 monotherapy were unsatisfactory. While allosteric inhibitor **47** potently inhibits the proliferation of tumour cell lines *in vivo* and *in vitro* even as a single agent^74^, accelerated EGFR dimerisation still affects its inhibitory activity. Therefore, to obtain better efficacy, the inhibitors occupying the ATP-binding pocket and allosteric pocket have significant inhibitory activity against both EGFR^L858R/T790M/C797S^ and EGFR^Del19/T790M/C797S^ while exhibiting high selectivity for EGFR^WT ^^125^. However, to distribute them in a larger space from the ATP binding site to the allosteric pocket, they tend to be large, so their bioavailability is low^38^. In addition, some fourth-generation EGFR-TKIs are currently in early clinical trials, and more data are needed to verify their safety and efficacy in the future^126^ (Figure 31).

Consequently, there is a need for the development of additional novel scaffolds as candidates for fourth-generation EGFR-TKIs. Readily available, structure-rich natural products present significant promise as a source of candidate drug compounds^127^^,^^128^ (Figure 31). Fakih and coworkers^129^ discovered that clathrin-A compounds have the potential to function as EGFR allosteric inhibitors. These natural compounds exhibit a different structure compared to EGFR-TKIs that target EGFR^C797S^. Moreover, the application of machine learning techniques for structural innovation can be highly beneficial^130^^,^^131^. Li and coworkers^132^ utilised structural descriptors as guidance during the modelling process and identified four exceptional compounds after virtually screening a dataset of approximately 50 000 compounds. This approach enhances the precision and efficiency of screening for novel EGFR-TKI scaffolds (Figure 31). Further optimisation of these compounds may yield an innovative structural framework for EGFR-TKIs (Figure 31).

While developing new scaffolds for fourth-generation EGFR-TKIs, explore other therapeutic pathways for triple mutations. Unlike traditional enzyme inhibitors that only inhibit the catalytic activity of the target enzyme, they induce degradation of their target proteins and are cleared *in vivo*; studies have shown that EGFR allosteric inhibitors such as EAI045, TQB3804, and brigatinib effectively inhibit and degrade various EGFR mutants^133^ (Figure 31). Recently, Zhang and coworkers^134^ developed and synthesised a potent and specific PROTAC degrader targeting the EGFR^Del19/T790M/C797S^ triple mutation.

Clinical trials have shown that combination therapy with EGFR-TKIs may be effective in patients with osimertinib resistance^135^^,^^136^ (Figure 31). The combination therapy strategy proposed by Cho and coworkers for osimertinib and its compound BI-4732 effectively targets EGFR mutations including C797S^137^; John et al.^138^ reported that patients with T790M and C797S mutations showed rapid clinical improvement after two weeks of combination therapy with gefitinib and osimertinib and no adverse drug events. In addition, other combination therapies involve drug combinations, such as the pairing of third-generation inhibitors with MEK inhibitors; osimertinib in combination with oxidative phosphorylation inhibitors, and other drugs.

In addition to combination therapy, multi-target drugs offer several advantages (Figure 31). It offers a broader range of activity while minimising the risk of potential drug interactions and side effects^139^. Cai et al.^140^ synthesised a multi-target compound, designated as compound **8**, which exhibits strong *in vitro* inhibitory activity against histone deacetylase (HDAC), EGFR, and human epidermal growth factor receptor 2 (HER2). This was achieved by incorporating HDAC inhibition into the pharmacophore of EGFR and HER2 inhibitors. Greene and coworkers^141^ developed a small kinase inhibitor, ER121. It demonstrates good tolerability when administered orally and exhibits significant inhibitory activity against EGFR^C797S^ and amplified ErbB2.

In clinical practice, according to the patient’s gene mutation profile, a personalised treatment plan is formulated, and the best treatment drug is selected based on the mutant subtype (cis/trans-C797S) to improve the efficacy^138^ (Figure 31).

In conclusion, this review primarily summarises the classification of EGFR-TKIs associated with EGFR^C797S^ mutations, their *in vitro* and *in vivo* inhibitory activities, and the mechanisms of action between the inhibitors and the active site. Additionally, it discusses the progression of drug molecules currently undergoing clinical studies. It is anticipated that this review will assist researchers in designing and developing effective EGFR-TKIs targeting the EGFR^C797S^ mutation by offering new insights.

## Data Availability

Not applicable.
